# Furthering Scientific Inquiry for Weight Loss Maintenance: Assessing the Psychological Processes Impacted by a Low intensity Technology-Assisted Intervention (NULevel Trial)

**DOI:** 10.1093/abm/kaae002

**Published:** 2024-02-23

**Authors:** Keven Joyal-Desmarais, Alexander J Rothman, Elizabeth H Evans, Vera Araújo-Soares, Falko F Sniehotta

**Affiliations:** School of Psychology, University of Leeds, Leeds, UK; Department of Psychology, University of Minnesota, Minneapolis, MN, USA; Department of Psychology, Durham University, Durham, UK; Department for Prevention of Cardiovascular and Metabolic Disease, Medical Faculty Mannheim, CPD, University of Heidelberg, Mannheim, Germany; Department for Public Health, Social and Preventive Medicine, Medical Faculty Mannheim, CPD, University of Heidelberg, Mannheim, Germany; NIHR Policy Research Unit Behavioural Science, Faculty of Medical Sciences, Newcastle University, Newcastle, UK

**Keywords:** Body weight, Weight loss, Health promotion, Randomized controlled trial, Obesity, Process study

## Abstract

**Background:**

NULevel was a randomized control trial to evaluate a technology-assisted weight loss maintenance (WLM) program in the UK. The program included: (a) a face-to-face goal-setting session; (b) an internet platform, a pedometer, and wirelessly connected scales to monitor and report diet, physical activity, and weight, and; (c) regular automated feedback delivered by mobile phone, tailored to participants’ progress. Components were designed to target psychological processes linked to weight-related behavior. Though intervention participants showed increased physical activity, there was no difference in WLM between the intervention and control groups after 12 months (Sniehotta FF, Evans EH, Sainsbury K, et al. Behavioural intervention for weight loss maintenance versus standard weight advice in adults with obesity: A randomized controlled trial in the UK (NULevel Trial). PLoS Med. 2019; 16(5):e1002793. doi:10.1371/journal.pmed.1002793). It is unclear whether the program failed to alter targeted psychological processes, or whether changes in these processes failed to influence WLM.

**Purpose:**

We evaluate whether the program influenced 16 prespecified psychological processes (e.g., self-efficacy and automaticity toward diet and physical activity), and whether these processes (at 6 months) were associated with successful WLM (at 12 months).

**Methods:**

288 adults who had previously lost weight were randomized to the intervention or control groups. The control group received wireless scales and standard advice via newsletters. Assessments occurred in person at 0, 6, and 12 months.

**Results:**

The intervention significantly altered 10 of the 16 psychological processes, compared with the control group. However, few processes were associated with WLM, leading to no significant indirect effects of the intervention via the processes on WLM.

**Conclusions:**

Changes in targeted processes were insufficient to support WLM. Future efforts may more closely examine the sequence of effects between processes, behavior, and WLM.

## Teaser

A 12-month weight-loss maintenance program successfully impacted psychological processes (e.g., a person’s confidence) tied to physical activity, healthy eating, and self-weighing. However, these effects did not translate to improved weight-loss maintenance.

To help manage health effects related to high body weight, it is beneficial for intervention strategies to be available that not only help people pursue weight loss goals, but also maintain weight loss over time. Yet, strategies that support weight loss maintenance (WLM) have proven difficult to identify—an issue that remains a key challenge in obesity management [1]. The NULevel trial tested a scalable, low-intensity, technology-assisted intervention to support WLM over a 12-month period [2]. Participants, who had an initial body mass index (BMI) ≥30 and then lost at least 5% of their bodyweight prior to entering the trial, experienced limited weight gain (mean = 1.8 kg) during the trial period; however, the rate of weight gain did not differ significantly between those randomized to the intervention or the control condition [3]. Why was the intervention not superior to the control condition? Did it fail to engage the psychological processes it was designed to target (e.g., self-efficacy, planning, and automaticity)? Were changes in these processes unrelated to WLM? Or might another explanation underlie these results? The present paper addresses these questions using data from the NULevel trial that tracked changes in a set of 16 targeted psychosocial constructs over the 12-month study period.

## Mapping an Intervention Effect for NULevel Using the Experimental Medicine Approach

**Fig. 1.**
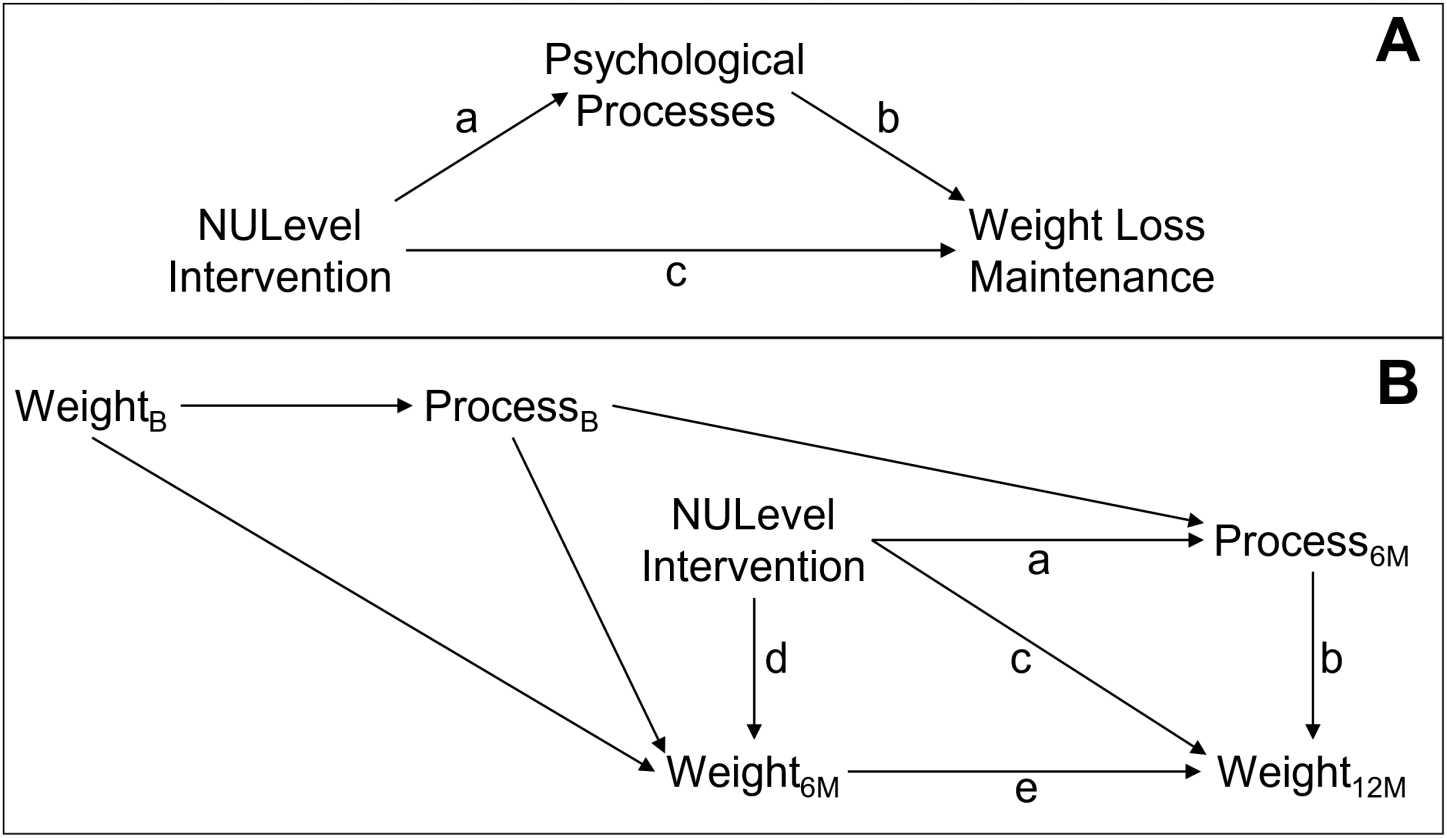
Theoretical models guiding our analyses. The upper panel (Panel A) outlines our project within the experimental medicine (EM) approach to health behavior change. The lower panel (Panel B) provides a directed acyclic graph (DAG) delineating how the variables in Panel A play are theorized to operate over the three time points of the NULevel trial. Panel B also represents a path diagram that matches the path analytic models computed in the current project. Path *a* captures the direct effect of the intervention on psychological processes at 6 months (6M). Path *b* captures the direct effect of the psychological process (measured at 6 months) on weight at 12 months (12M). The composite Path *a* × *b* captures the indirect effect of the intervention on weight at 12 months (12M) attributed to changes on the process at 6 months (6M; accounting for paths *c* and *d × e*). *Path c* captures the direct effect of the intervention on weight at 12 months (12M; accounting for paths *a* × *b* and *d* × *e*). Path *d* captures the direct effect of the intervention on weight at 6 months (6M). Path *e* captures the direct effect of weight at 6 months (6M) on weight at 12 months (12M). Finally, the composite Path *d × e* captures the indirect effect of the intervention on weight at 12 months (12M) attributed to changes on weight at 6 months (6M). Panel B also shows the theorized roles of weight and the psychological processes at baseline (B) as they relate to other variables in the model.

The effectiveness of behavioral interventions relies on their ability to elicit change in one or more target constructs (e.g., self-efficacy, planning) that in turn change a desired health outcome (e.g., body weight). This framework is captured by a series of paths that comprise the Experimental Medicine approach (see Fig. 1A)—one path links the intervention to the targeted construct(s) (i.e., the *engagement* path) and the other links the targeted construct(s) to the primary outcome (i.e., the *validity* path [4, 5]). Elucidating the operation of these paths is a key part of intervention process evaluation as it can clarify *why* an intervention is or is not effective.

The NULevel intervention was grounded on the premise that WLM is a function of sustained patterns of dietary behavior, physical activity, and weight monitoring. To promote such sustained behaviors, the program was developed in line with a systematic review of theories of behavioral maintenance [6], with a particular focus on self-regulation theories [7, 8]—which we observed to underpin previously successful WLM interventions [9]. Such theories emphasize that a person’s success at maintaining behaviors (e.g., greater physical activity, improved diet) generally depends on their ability to confidently enact self-regulation strategies (e.g., self-monitoring, action planning), particularly in the face of challenges (e.g., using coping planning to resist temptations). The theories further emphasize the importance of sustaining motivation throughout goal pursuit (e.g., such as by feeling satisfaction with obtained changes) and of maintaining adequate regulatory resources (e.g., automatizing behaviors to conserve energy [6]).

The current intervention engaged with the themes above by targeting five classes of constructs: (a) people’s perceptions of control over, and confidence in, engaging in weight-related behaviors (i.e., physical activity, dietary change, and self-weighing); (b) their energy levels for these behaviors; (c) their ability to develop and implement plans to manage these behaviors; (d) the degree to which these behaviors become automated; and (e) their satisfaction with the outcomes afforded by changes in their weight. To target these constructs, intervention participants engaged in an initial goal-setting session and used an internet-based platform to track their diet, physical activity, and weight. Participants were also given a pedometer and a wireless scale, with instructions to weigh themselves daily. Feedback via text messages and phone were then incorporated to further promote the psychological variables [2, 3].

Leveraging measures of the targeted constructs at baseline, 6 months, and 12 months, we examined two exploratory questions: (a) During the first 6 months of the trial, did the intervention elicit greater change in the targeted constructs than did the control condition?, and (b) To what extent did initial changes in the targeted constructs lead to change in weight during the last 6 months of the trial? Answers to the first question will indicate whether the intervention engaged the constructs it was designed to target, and answers to the second question will indicate whether the targeted constructs were successful predictors of weight maintenance.

## Methods

The NULevel trial was a registered two-armed randomized controlled superiority trial [10]. The analyses making up the current work were also preregistered prior to conducting analyses [11], outlining measures to be used, how measures were to be scored, and analyses to be conducted. The current work reports all preregistered analyses without deviation.

### Sample

Participants were recruited in England between April 2014 and May 2015. Participants were 288 adults (≥18 years) who (a) had lost 5% or more of their weight in the 12 months preceding the trial and (b) had had a BMI equal to or greater than 30 kg/m^2^ (or ≥28 kg/m^2^ for individuals of South Asian descent) within 12 months preceding the NULevel trial. Participants had a mean age of 41.8 years, a mean baseline weight of 85.6 kg, and a mean baseline BMI of 30.9 kg/m^2^. Additionally, 77% identified as female (33% as male), about half (49%) had a household income of £40,000 or above, and most were employed full (61%) or part-time (17%). Overall, 144 participants were randomly allocated to the intervention group and 144 to the control group. Further details on recruitment, inclusion and exclusion criteria, randomization, and participant demographics were previously reported [2, 3].

### NULevel Arms

Detailed descriptions of the intervention design and control groups are reported elsewhere [2, 3]. In brief, the *intervention* lasted 12 months and consisted of elements such as a face-to-face goal-setting session, along with access to an internet-based platform, a pedometer, and a wirelessly connected scale—which participants were instructed to use to regularly monitor and report their diet, physical activity, and weight. The intervention group was given regular feedback tailored to people’s goal progress, delivered via short message service (SMS) messages with links to mobile internet content. A “traffic light” system was also set up to trigger contacts (by phone) with the intervention team based on participant weight regain. This system allowed the intervention to be less intensive when participants’ weight was stable.

Intervention elements were designed to target processes tied to WLM behavior change, including the 16 variables in Table 1. For example, during the face-to-face sessions, participants made plans to engage in physical activity and healthy eating, and generated strategies to overcome barriers–engaging in action planning and coping planning, while bolstering their confidence and capability in enacting the behaviors. SMS messages were used to encourage further planning and goal regulation, prompt behaviors and habit-formation (automaticity), and to highlight past and current successes (reinforcing satisfaction with outcomes).

**Table 1:** Summary of the 16 Psychological Process Variables Measured

Process variable	Description	Example item (number of items)	Scaling	*α*_s_
01. Satisfaction with changes	Satisfaction with weight change related outcomes (e.g., self-esteem, fit in clothes).	How satisfied are you with [change in] your self-esteem? (11)	1 = unhappy/dissatisfied; 3 = happy/satisfied	0.91 0.94
02. PBC: Healthy eating	Confidence in and perceived ease of eating healthy foods in moderation.	How confident are you in your ability to eat healthy foods in moderation? (2)	1 = Not confident; 7 = confident	0.75 0.86
03. PBC: Physical activity	Confidence in and perceived ease of being physically active every day.	How confident are you in your ability to be physically active every day? (2)	1 = Not confident; 7 = confident	0.83 0.86
04. Confidence: Weight loss	Confidence in ability to lose weight.	How confident are you in your ability to lose weight? (1)	1 = Not confident; 7 = confident	n/a
05. Confidence: WLM	Confidence in ability to maintain weight loss.	How confident are you in your ability to maintain weight loss? (1)	1 = Not confident; 7 = confident	n/a
06. SE: Emotional eating	Perceived capacity to avoid unhealthy foods when experiencing negative affect	I can resist eating unhealthy food when I am anxious. (3)	1 = False; 4 = True	0.91 0.95
07. SE: Unhealthy food context	Perceived capacity to avoid unhealthy foods in the face of contextual barriers.	I can resist eating unhealthy food even when I am at a party. (10)	1 = False; 4 = True	0.86 0.93
08. SE: Physical activity barriers	Perceived capacity to engage in physical activity even in the face of barriers.	I will be physically active every day even when I am sad. (12)	1 = False; 4 = True	0.95 0.96
09. Action planning: physical activity	Making concrete plans to be physically active.	I have made a detailed plan regarding when to be physically active. (3)	1 = Totally disagree; 4 = Totally agree	0.95 0.96
10. Action planning: Healthy eating	Making concrete plans to make healthy food choices.	I have made a detailed plan regarding when to make healthy food choices. (3)	1 = Totally disagree; 4 = Totally agree	0.96 0.97
11. Coping planning: Physical activity	Making plans to overcome barriers against physical activity.	I have made a detailed plan regarding how to keep on being physically active in difficult situations. (3)	1 = Totally disagree; 4 = Totally agree	0.96 0.96
12. Coping planning: Healthy eating	Planning to overcome barriers and temptations against healthy eating.	I have made a detailed plan regarding what to do if I’m tempted by unhealthy foods. (4)	1 = Totally disagree; 4 = Totally agree	0.94 0.95
13. Automaticity: Healthy eating	Automaticity of healthy eating behavior.	Making healthy food choices is something I do automatically. (5)	1 = False; 4 = True	0.93 0.95
14. utomaticity: Physical activity	Automaticity of doing physical activity behavior.	Being physically active is something I do without thinking. (5)	1 = False; 4 = True	0.96 0.97
15. Automaticity: Self-weighing	Automaticity of engaging in self-weighing behavior.	Weighing myself is something I do without having to consciously remember. (5)	1 = False; 4 = True	0.95 0.97
16. Energy and drive	Feeling energetic and driven, as opposed to exhausted and fatigued.	Over the last 2 weeks I have felt full of vitality. (12)	1 = None of the time; 5 = All of the time	0.93 0.94

*α*_*s*_ lowest and highest Standardized Reliability Alphas (for 3+ item scales) or *r* (for 2 items scales) across the three time points of the study; *n/a* not applicable; *PBC* perceived behavioral control; *Scaling* example anchors used with items, reflecting the minimum/maximum possible scores; *SE* self-efficacy; *WLM* weight loss maintenance.

The *control* group received a wirelessly connected scale—which they could use to monitor their weight, though they were not instructed to do so—and standard National Health Service lifestyle advice delivered using an SMS link once every three months. Both groups were aware that data from the wireless scales would be sent to the research team.

### Variables/Measures

Each variable was measured at three time points: baseline (B; pre-intervention), 6 months into the intervention (6M), and at 12 months, immediately upon completing the intervention (12M; post-intervention).

### Weight

The primary outcome was body weight, in kilograms (kg) assessed at 12 m. At B and 12M, weight was measured using a digital portable scale by research staff who were blind to group allocations. At 6M participants self-reported their weight, and a subset of participants (*N* = 68) also provided self-reports at 12M (in addition to measurements by staff). The self-reports and objective measures at 12M were correlated at *r* = 0.998, and most participants reported weights equal to that obtained via objective measurement. This suggests that participants’ self-reports were highly accurate (see Supplementary Files for more details). Thus, the current project used objective weight measurements at B and 12M, supplemented by self-reports at 6M.

### 16 Psychological Processes

At all three time points, participants completed measures of 16 psychological processes that have previously been tied to weight-related behaviors. These included measures of participants’ *satisfaction with* (*weight-related) changes* (using the Weight Outcomes Satisfaction Scale [12]); *perceived behavioral control* (PBC), *self-efficacy* (SE), and *confidence* toward healthy eating, physical activity, weight loss and WLM (using items adapted from previous scales [13, 14]); *action planning* and *coping planning* (using items adapted from previous scales [15]); *automaticity* of healthy eating, physical activity and self-weighing (using items adapted from the Self-Report Behavioral Automaticity Index [16, 17]), and; participants’ *energy and drive* (using a measure developed for the current study, previously referred to as “ego depletion” [3]). Table 1 provides descriptions and example items for each process, and the Supplementary Materials provide additional details on each measure, including the full list of items, notes on the development of each measure, psychometric results (e.g., confirmatory factor analyses [CFA]), and descriptive statistics (e.g., means, standard deviations) by time point. Scales had high reliability (alphas usually above 0.90), and CFAs found all items to consistently and significantly load onto their respective factors above 0.40.

For each multi-item measure, a mean score was calculated using available responses. To ease interpretation, each scale was scored such that higher numbers reflected a more positive disposition to engage in weight-related behaviors (higher score = higher satisfaction, SE, PBC, etc.). Apart from this adjustment, analyses used raw scores, without transformations.

### Analyses

Path analytical models were specified according to Panel B of Fig. 1 and computed individually for each of the 16 process variables in Table 1. Our primary inferences focus on three pathways from Fig. 1. Path *a* captures the direct effect of the intervention on the psychological processes at 6 months (6M), accounting for earlier levels of the processes at baseline. Path *b* captures the direct effect of the processes (measured at 6 months) on weight at 12 months (12M), accounting for past weight. Path *a × b* captures the indirect effect of the intervention on weight at 12 months (12M) attributed to changes on the processes at 6 months (6M)—accounting for direct effects of the intervention on 12M weight, and indirect effects on 12M weight through 6M weight. Path *a × b* was used to evaluate mediation [18, 19]. Analyses were conducted using the *lavaan* [20] package in *R* [21]. To draw inferences, we used null hypothesis significance testing with an alpha level of 0.05. We constructed 95% confidence intervals (CI) using a bias-corrected bootstrap percentile method with 10,000 bootstrap samples. Full information maximum likelihood was used to account for missing data [22–24]. Our analysis script is available at: https://osf.io/5qnu7/.

## Results

### Weight per Time Point

There was no difference in weight maintenance across the two conditions, reproducing past findings [3]. In the control group, mean weight [and 95% CI] at each time point was: B = 85.51 kg [82.92, 88.11]; 6M = 83.92 kg [80.93, 86.91]; and 12M = 86.67 kg [83.63, 89.71]. In the intervention group, the weights at each time point were: B = 85.58 kg [82.71, 88.44]; 6M = 84.63 kg [80.99, 88.26]; and 12M = 87.03 kg [83.81, 90.26].

### Change in Processes from B to 6M

To contextualize our findings, Table 2 provides the results of paired-sample *t*-tests evaluating the degree to which participants’ scores on each psychological process changed from B to 6M, expressed as Cohen’s *d*, by study group. Overall, the control group showed notable declines in many processes from B to 6M (10 of 16 variables decreased by *d* = −0.21 to −0.62). In contrast, declines were generally less pronounced, often even absent, in the intervention group, which even showed significant increases in three process variables (increases of *d* = 0.23 to 0.51).

**Table 2:** Summary of Results with Path Analytic Models Specified According to Fig. 1 (Panel B)

Process variable	Descriptive means		Process change (*t*-tests: B → 6M)				Path analytic findings					
	Control	Intervention	Control		Intervention		Path *a*		Path *b*		Path *a × b*	
	B (6M)	B (6M)	*d*	sig.	*d*	sig.	β	sig.	β	sig.	β	sig.
01. Satisfaction with changes	2.63 (2.22)	2.65 (2.47)	**−0.62**	***	**−0.34**	***	**0.18**	**	−0.02		0.00	
02. PBC: Healthy eating	5.10 (4.82)	5.41 (5.60)	**−0.26**	**	0.11		**0.21**	***	−0.09	^t^	**−**0.02	^t^
03. PBC: Physical activity	4.63 (4.29)	4.70 (4.56)	**−0.21**	*	**−**0.11		0.07		**−0.05**	*	0.00	
04. Confidence: Weight loss	5.15 (4.33)	5.51 (5.00)	**−0.56**	***	**−0.31**	***	**0.16**	**	**−**0.04		**−**0.01	
05. Confidence: WLM	4.16 (4.08)	4.30 (5.20)	−0.09		**0.51**	***	**0.30**	***	**−**0.04		**−**0.01	
06. SE: Emotional eating	2.44 (2.56)	2.76 (2.61)	0.08		**−**0.14		**−**0.04		**−**0.01		0.00	
07. SE: Unhealthy food context	2.94 (2.75)	2.94 (2.94)	**−0.35**	***	0.00		**0.15**	**	**−**0.02		0.00	
08. SE: Physical activity barriers	2.92 (2.70)	2.96 (2.80)	**−0.34**	***	**−0.21**	*	0.05		**−**0.05		0.00	
09. Action planning: Physical activity	2.91 (2.59)	2.83 (2.66)	**−0.33**	***	**−**0.11		0.07		0.00		0.00	
10. Action planning: Healthy eating	3.15 (2.78)	3.30 (3.20)	**−0.43**	***	**−**0.12		**0.20**	***	**−**0.02		0.00	
11. Coping planning: Physical activity	2.17 (2.06)	2.22 (2.28)	**−**0.16	^t^	0.07		0.11	^t^	**−**0.03		0.00	
12. Coping planning: Healthy eating	2.62 (2.43)	2.81 (2.85)	**−0.24**	*	0.03		**0.19**	**	**−**0.03		**−**0.01	
13. Automaticity: Healthy eating	2.60 (2.63)	2.74 (2.94)	0.05		**0.23**	*	**0.14**	*	**−0.14**	*	**−**0.02	^t^
14. Automaticity: Physical activity	2.36 (2.36)	2.42 (2.49)	0.02		0.10		0.05		**−**0.05		0.00	
15. Automaticity: Self-weighing	2.68 (2.67)	2.72 (3.19)	0.00		**0.41**	***	**0.25**	***	**−**0.01		0.00	
16. Energy and drive	3.39 (3.15)	3.51 (3.24)	**−0.42**	***	**−0.34**	***	0.02		**−0.06**	*	0.00	

*β* standardized coefficient; *6M* 6-month follow-up; *B* baseline; *d* Cohen’s *d*; *PBC* perceived behavioral control; *SE* self-efficacy; *sig*. significance level; *WLM* weight loss maintenance. **Bold font** indicates significance at *p* < .05.

^a^See Supplementary Materials (Table S49) for full *t*-test results (e.g., for unstandardized difference scores, 95% CIs, and exact *p*-values).

^b^See Supplementary Materials (Tables S33–S48) for full path analytic results (e.g., for unstandardized effects, standard errors, 95% CIs, exact *p*-values, fit indices).

***p < .001; **p < .01; *p < .05; ^t^*p* < .10.

### Inferential Tests of the Role of Each Process

Table 2 presents the results of path analytic models, specified in line with Fig. 1 (Panel B), for each of the 16 psychological processes. We present standardized coefficients for Path *a*, Path *b*, and the indirect effect of Path *a × b*. For these paths (and other paths in Fig. 1), the Supplementary Materials additionally provide unstandardized regression coefficients, standard errors, 95% CIs, exact *p*-values, fit indices, and coefficients of determination (*R*^2^). Path *a* was significant for 9 of the 16 processes; indicating that compared with the control condition, the intervention was better able to sustain (i.e., avoid declines in) or increase people’s levels of satisfaction, PBC (for healthy eating), confidence (toward weight loss and WLM), SE (in the face of unhealthy food), action and coping planning (for healthy eating), and automaticity (for healthy eating and self-weighing). Yet, there was limited evidence that the psychological processes were associated with weight change; Path *b* was significant in only three instances (PBC for physical activity; automaticity for healthy eating; energy and drive), and the mediational Path *a × b* was never significant.

## Discussion

The degree to which behavioral interventions are successful often depends on their ability to change psychological processes, and, in turn, on the degree to which changes in those processes are sustained and impact people’s behaviors [4, 5]. In this project, we conducted a process analysis of the NULevel trial to identify why the intervention did not outperform a control group to impact WLM. To do this, we examined the influence of the intervention on 16 psychological processes targeted by the intervention (Fig. 1, Path *a*), the degree to which change in these processes was associated with weight change over time (Path *b*), and the extent to which these two forces jointly impacted weight change outcomes (Path *a × b*).

Our analyses revealed several patterns. First, the intervention appeared to alter how most of the psychological processes it targeted changed over time (Path *a*). Generally, participants in the control group showed substantial decline on most processes over time (e.g., in satisfaction, weight loss confidence, and action planning), but this decline was attenuated within the intervention condition. For a few processes (e.g., confidence in WLM, automaticity), participants in the intervention condition showed increased scores over time, relative to the control group. Despite the intervention’s ability to stabilize or promote psychological processes, we found limited support for higher scores on these processes being associated with successful WLM (Path *b*) over time within the context of this trial. Consequently, the operation of the intervention on weight outcomes through these processes (the composite Path *a × b*) was never significant. This occurred despite an observation that within any given time point (i.e., cross-sectionally), the majority of processes were significantly correlated with weight.

If the intervention successfully engaged the psychological processes it targeted, then why did these changes not lead to greater WLM? One explanation would be that, though the processes are amenable to influence via intervention, they may not be reliable determinants of WLM. Given that these processes were selected based on their theoretical relevance to behavioral maintenance [6–8], replicating our findings in other interventions would be necessary before seeking to dismiss the processes. This is particularly so as other explanations could also account for our findings. In particular, we note that both the intervention *and* the control group performed well, each gaining an average of less than 1.5 kg over the 12 months of the trial. It is possible that the provision of elements such as a wireless scale to the control group, along with the knowledge that data from these scales would be accessible to the research team, was sufficient to encourage certain processes (e.g., self-monitoring) that supported WLM [25–27]. This possibility is consistent with the observation that, during the trial, control group participants frequently engaged in self-weighing, averaging twice a week [3]. If so, this could have attenuated our chances of finding the intervention to be favorable relative to the control (though the link between self-weighing and WLM remains to be verified). Similarly, attenuated variability in WLM across the groups may have interfered with observing associations over time between processes and weight change (Path *b*).

A second possible explanation for our findings is that although the intervention impacted WLM processes, the effects may have been too small to differentially affect WLM. Consistent with this point, when the effects of the NULevel intervention on processes at 6 months were present (i.e., statistically significant for 9 of 16 variables) these could only be qualified as small-to-medium in magnitude [28]. Perhaps more important, however, is that the processes themselves are thought of as direct precursors to behavior, not weight change, and it is behavior (not the processes themselves) that ultimately affects weight. Thus, the lack of substantial effects from the processes to WLM could reflect either a failure of the processes to impact behavior, or that changes in behavior had only a modest impact on weight. For example, the association between weight and change in behaviors such as physical activity, diet, and self-weighing is known to be variable [26, 29–32]. Such a dynamic could considerably attenuate the overall impact of targeting processes to alter behavior to then impact weight. The lack of a behavioral analysis is an important limitation of the current study, and should be a major focus for future work. Additionally, inferences are limited by the time frames that were used to assess the constructs of interest. For instance, it is possible that assessing weight at 6-month intervals was too infrequent, and that using shorter time intervals (e.g., weekly or monthly) would reveal more consistent associations between the proposed processes and weight. Likewise, many of the process measures were brief in scope, and it may be that clearer mediational patterns would emerge by using more comprehensive and frequent measures of the processes.

Ultimately, establishing whether the processes elicit change in desired WLM behaviors (healthier diet, physical activity), and how reliably such behaviors determine WLM, will not only provide further insights on the NULevel trial, but will inform future intervention efforts as well. For instance, if both pathways hold, but only show modest effects, it could be that the intensity of the intervention was too low, and that stronger impacts on the 16 psychological processes should be sought. In the current trial, most of the significant effects were also related to the intervention dampening reductions in the processes otherwise seen in the control group rather than bolstering them in the intervention group. If processes need to be maintained above a certain threshold level to have an impact on behavior and WLM [33], then the intervention might need to achieve stronger bolstering effects on the processes to achieve WLM. As the intervention was *designed* to be low-intensity to facilitate scalability [2, 3], implementing methods to augment the strength of the intervention could remain promising. This could be by increasing the “dosage” of the behavior change techniques (BCTs) [34] used, or by tailoring their use to better account for people’s needs/preferences [35].

In contrast, if there is no effect between the processes and the behaviors, or between behaviors and WLM, then this would suggest targeting other psychological processes than assessed in the current study (using appropriate BCTs) [34], or more specific forms of behaviors that have more consistent effects on WLM. Both of these possibilities offer substantial avenues for future intervention work. In paying attention to the effects between processes, behaviors, and WLM, it may also be useful to examine the degree to which these vary across groups of participants, as it is unlikely all participants experience benefits via the same mechanisms of action. Such information would be invaluable to further personalize future WLM interventions.

## Supplementary Material

kaae002_suppl_Supplementary_Material
