# Supplementary material for: Furthering Scientific Inquiry for Weight Loss Maintenance: Assessing the Psychological Processes Impacted by a Low intensity Technology-Assisted Intervention (NULevel Trial)
Source: Ann Behav Med. 2024 Feb 23;58(4):296–303. doi: 10.1093/abm/kaae002 (PMC10928833; doi:10.1093/abm/kaae002)
Supplement: kaae002_suppl_Supplementary_Material [file kaae002_suppl_supplementary_material.pdf]

Electronic Supplemental Material (Text)

Click here to access/download;Electronic Supplemental Material (Text);Supplemental Files.docx

— SUPPLEMENTAL FILES —

Furthering Scientific Inquiry for Weight Loss Maintenance:

Assessing the Psychological Processes Impacted by a Low intensity Technology-Assisted

Intervention (NULevel Trial)

Table of Contents

1. Process Measures – Summary Table ... 3
2. Process Measures – Full Details (Items, Confirmatory Factor Analyses, Alphas, Descriptive Statistics) ... 4
2.01. Satisfaction with Changes ... 4
2.02. Perceived Behavioral Control (PBC) Measures: Healthy Eating & Physical Activity. ... 6
2.03. Confidence Measures: Weight Loss and Weight Loss Maintenance (WLM). ... 8
2.04. Self-Efficacy (SE): Emotional Eating, Unhealthy Food Context, Physical Activity Barriers. ... 9
2.05. Action Planning & Coping Planning: Healthy Eating and Physical Activity. ... 13
2.06. Automaticity: Healthy Eating, Physical Activity, and Self-Weighing. ... 16
2.07. Energy and Drive. ... 18
3. Weight Assessment (Measurement Info, Correlations, and Descriptive Statistics) ... 21
4. Detailed Results from Path Analyses ... 23
4.01. Results: Path a ... 24
4.02. Results: Path b ... 25
4.03. Results: Path c ... 26
4.04. Results: Path d ... 27
4.05. Results: Path e ... 28
4.06. Results: Path f ... 29
4.07. Results: Path g ... 30
4.08. Results: Path h ... 31
4.09. Results: Path i ... 32
4.10. Results: Composite Path a*b ... 33
4.11. Results: Composite Path d*e ... 34
4.12. Results: Composite Path “decab” [(d*e) + c + (a*b)] ... 35
4.13. Results: Composite Path “ibhe” [(i*b) + (h*e)] ... 36
4.14. Results: Composite Path “gefhe” [(g*e + f*h*e)] ... 37
4.15. Results: Composite Path “gefhefib” [(g*e + f*h*e + f*i*b)] ... 38
4.16. Results: Fit Indices and R^2 Values for Each Path Analytic Model. ... 39
5. Results: *T-T*ests and Descriptives of the Change in Each Process from Time 1 to Time 2 ... 40
6. References ... 42

1. Process Measures – Summary Table

Table S1. Summary Overview of Measures for the 16 Psychological Process.

Process Variable	Description	Example Item (Number of Items)	Scaling	α_s
**01. Satisfaction with Changes**	Satisfaction with weight change related outcomes (e.g., self-esteem, fit in clothes).	How satisfied are you with [change in] your self-esteem? (11)	1=unhappy/dissatisfied; 3= happy/satisfied	.91 .94
**02. PBC: Healthy Eating**	Confidence in and perceived ease of eating healthy foods in moderation.	How confident are you in your ability to eat healthy foods in moderation? (2)	1= Not confident; 7 = confident	.75 .86
**03. PBC: Physical Activity**	Confidence in and perceived ease of being physically active every day.	How confident are you in your ability to be physically active every day? (2)	1= Not confident; 7 = confident	.83 .86
**04. Confidence: Weight Loss**	Confidence in ability to lose weight.	How confident are you in your ability to lose weight? (1)	1= Not confident; 7 = confident	n/a
**05. Confidence: WLM**	Confidence in ability to maintain weight loss.	How confident are you in your ability to maintain weight loss? (1)	1= Not confident; 7 = confident	n/a
**06. SE: Emotional Eating**	Perceived capacity to avoid unhealthy foods when experiencing negative affect	I can resist eating unhealthy food when I am anxious. (3)	1= False; 4 = True	.91 .95
**07. SE: Unhealthy Food Context**	Perceived capacity to avoid unhealthy foods in the face of contextual barriers.	I can resist eating unhealthy food even when I am at a party. (10)	1= False; 4 = True	.86 .93
**08. SE: Physical Activity Barriers**	Perceived capacity to engage in physical activity even in the face of barriers.	I will be physically active every day even when I am sad. (12)	1= False; 4 = True	.95 .96
**09. Action Planning: Physical Activity**	Making concrete plans to be physically active.	I have made a detailed plan regarding when to be physically active. (3)	1=Totally disagree; 4 = Totally agree	.95 .96
**10. Action Planning: Healthy Eating**	Making concrete plans to make healthy food choices.	I have made a detailed plan regarding when to make healthy food choices. (3)	1=Totally disagree; 4 = Totally agree	.96 .97
**11. Coping Planning: Physical Activity**	Making plans to overcome barriers against physical activity.	I have made a detailed plan regarding how to keep on being physically active in difficult situations. (3)	1=Totally disagree; 4 = Totally agree	.96 .96
**12. Coping Planning: Healthy Eating**	Planning to overcome barriers and temptations against healthy eating.	I have made a detailed plan regarding what to do if I'm tempted by unhealthy foods. (4)	1=Totally disagree; 4 = Totally agree	.94 .95
**13. Automaticity: Healthy Eating**	Automaticity of healthy eating.	Making healthy food choices is something I do automatically. (5)	1= False; 4 = True	.93 .95
**14. Automaticity: Physical Activity**	Automaticity of doing physical activity.	Being physically active is something I do without thinking. (5)	1= False; 4 = True	.96 .97
**15. Automaticity: Self-Weighing**	Automaticity of engaging in self-weighing.	Weighing myself is something I do without having to consciously remember. (5)	1= False; 4 = True	.95 .97
**16. Energy and Drive**	Feeling energetic and driven, as opposed to exhausted and fatigued.	Over the last 2 weeks I have felt full of vitality. (12)	1=None of the time; 5 All of the time	.93 .94

Notes. PBC = Perceived Behavioral Control; WLM = Weight Loss Maintenance; SE = Self-Efficacy; Scaling = example anchors used with items, reflecting the minimum/maximum possible scores; α_s = Lowest and highest Standardized Reliability Alphas (for 3+ item scales) or *r* (for 2 items scales) across the three time points of the study; n/a = not applicable. This table replicates Table 1 from the manuscript to ease referencing when consulting the supplemental file.

2. Process Measures – Full Details (Items, Confirmatory Factor Analyses, Alphas, Descriptive Statistics)

2.01. Satisfaction with Changes

Notes on scale and scale construction: Items come from the "satisfaction" and "satisfaction with the changes afforded by weight loss" scales used by Finch and colleagues in 2005 [1]. One item asked participants about their satisfaction towards their weight change over the preceding 12 months on a 5-point response scale, whereas the other 10 items asked about satisfaction about a variety of outcomes on a 3-point response scale. The original authors of the scale treated the former item separately from the other items. Prior to our registration, we evaluated two measurement models. One that separated the general item on satisfaction on weight change from the others, whereas the other model combined all 11 items. Including the item consistently led to a model with better fit indices and higher reliability alphas across all three time points. The item also showed a pattern of bivariate correlations to the other items that was similar to the pattern of bivariate correlations that any other item within the scale showed to the other items. Consequently, it did not appear as though the item was capturing a facet of satisfaction that was very distinct from the type of satisfaction captured overall by the other items. Therefore, we registered and conducted our primary analyses including this item within the set (rather than excluding it), leading this measure to be comprised of the 11 items in Table S2. Table S3 provides confirmatory factor analysis (CFA) loadings for the items across all time points, along for the scale's standardized reliability alpha at all three time points. Table S4 provides descriptive statistics for the scale across all three time points.

Table S2. *Items Used to Measure Satisfaction with Changes (11 items).*

Items	Anchors
How satisfied are you with the amount of weight you have lost and/or gained during the past 12 months?[a]	1 = very satisfied; 5 = very dissatisfied

How satisfied are you with this change/no change in terms of…your self-control?	1=happy/satisfied; 3=unhappy/dissatisfied
How satisfied are you with this change/no change in terms of…your self-esteem?	1=happy/satisfied; 3=unhappy/dissatisfied
How satisfied are you with this change/no change in terms of…your attractiveness?	1=happy/satisfied; 3=unhappy/dissatisfied
How satisfied are you with this change/no change in terms of…the fit of your clothes?	1=happy/satisfied; 3=unhappy/dissatisfied
How satisfied are you with this change/no change in terms of…any feelings of discomfort?	1=happy/satisfied; 3=unhappy/dissatisfied
How satisfied are you with this change/no change in terms of…the perceived risk to your health?	1=happy/satisfied; 3=unhappy/dissatisfied
How satisfied are you with this change/no change in terms of…your social life?	1=happy/satisfied; 3=unhappy/dissatisfied
How satisfied are you with this change/no change in terms of…negative judgements on your weight?	1=happy/satisfied; 3=unhappy/dissatisfied
How satisfied are you with this change/no change in terms of…positive feedback from others?	1=happy/satisfied; 3=unhappy/dissatisfied
How satisfied are you with this change/no change in terms of…negative feedback from others?	1=happy/satisfied; 3=unhappy/dissatisfied

[a]In taking the mean of items in this scale, scores on this first item were rescaled to be comparable to other scale items (i.e., be between 1-3).

Table S3. *Confirmatory Factor Analytic & Standardized Alpha Results for Satisfaction with Changes Measure.*

**How satisfied are you with this change/no change in terms of…**	**Standardized Factor Loadings**		
	**T1**	**T2**	**T3**
your self-control?	.61	.80	.76
your self-esteem?	.83	.89	.90
your attractiveness?	.75	.86	.85
the fit of your clothes?	.79	.89	.88
any feelings of discomfort?	.68	.79	.80
the perceived risk to your health?	.74	.81	.83
your social life?	.63	.62	.61
negative judgements on your weight?	.69	.73	.72
positive feedback from others?	.56	.79	.79
negative feedback from others?	.57	.53	.58
How satisfied are you with the amount of weight you have lost and/or gained during the past 12 months?	.78	.84	.83
**Standardized Reliability Alpha**	.91	.94	.94

Table S4. Descriptive Statistics per Time Point for Satisfaction with Changes Measure.

Time Point	Descriptive Statistic							
	**N**	**Mean**	**SD**	**Median**	**Min**	**Max**	**Skew**	**Kurtosis**
Time 1	288	2.64	0.51	2.82	1	3.18	-1.38	1.27
Time 2	232	2.34	0.67	2.55	1	3.20	-0.43	-1.18
Time 3	253	2.22	0.68	2.27	1	3.18	-0.15	-1.39

Notes. N = number of responses per time point; SD = Standard Deviation.

2.02. Perceived Behavioral Control (PBC) Measures: Healthy Eating & Physical Activity.

Notes on scale and scale construction: Items for the perceived behavioral control (PBC), self-efficacy (SE) and confidence measures were adapted from previous scales [2,3]. Exploratory Factor Analyses (EFA) along with an examination of correlation matrices across the first two time points were conducted on these items to examine how they should be partitioned into scales. From these analyses, we determined that PBC items should be separated into two scales depending on whether they focused on PBC towards healthy eating vs. physical activity (i.e., Table S5 vs. Table S6). Items within PBC scales were strongly correlated (r = .75 to 86), whereas items between the PBC scales were not (r = 35 to 42). We then conducted CFAs using Time 3 data to confirm this factor structure. Table S7 presents factor loadings for CFA models across the time points. Table S8 provides descriptive statistics.

Table S5. *Items Used to Measure Perceived Behavioral Control: Healthy Eating (2 items)*

Items	Anchors
How confident are you in your ability to... eat healthy foods in moderation?	1= Not confident; 7 = confident
How easy do you (think you will*) find it to... eat healthy foods in moderation?	1 = very difficult; 7 = very easy

Table S6. *Items Used to Measure Perceived Behavioral Control: Physical Activity (2 items)*

**Items**	**Anchors**
How confident are you in your ability to... be physically active every day?	1= Not confident; 7 = confident
How easy do you (think you will*) find it to... be physically active every day?	1 = very difficult; 7 = very easy

Table S7. *Confirmatory Factor Analytic & Correlations for the Perceived Behavioral Control Measures.*

**Items**	**Standardized Factor Loadings**[1]		
	**T1**	**T2**	**T3**
**PBC: Healthy Eating**			
How confident are you in your ability to... eat healthy foods in moderation?	.88	.98	.87
How easy do you (think you will*) find it to... eat healthy foods in moderation?	.85	.88	.94
**PBC: Physical Activity**			
How confident are you in your ability to... be physically active every day?	.94	.96	.92
How easy do you (think you will*) find it to... be physically active every day?	.89	.90	.93
**Correlations between items**	T1	T2	T3
PBC: Healthy Eating	.75	.86	.81
PBC: Physical Activity	.83	.86	.86

[1]Loadings were calculated at each time point from a single CFA model that included all seven of the PBC, Confidence, and SE measures.

Table S8. *Descriptive Statistics per Time Point for PBC Variables.*

**Time Point**	**Descriptive Statistic**							
	**N**	**Mean**	**SD**	**Median**	**Min**	**Max**	**Skew**	**Kurtosis**
**PBC: Healthy Eating**								
Time 1	286	5.25	1.19	5.0	1	7	-.42	-.04
Time 2	231	5.21	1.41	5.5	1	7	-.70	-.02
Time 3	253	5.21	1.46	5.5	1	7	-.75	.13
**PBC: Physical Activity**								
Time 1	286	4.67	1.50	5.0	1	7	-.25	-.69
Time 2	231	4.43	1.60	4.5	1	7	-.15	-.81
Time 3	253	4.65	1.75	5.0	1	7	-.40	-.79

Notes. N = number of responses per time point; SD = Standard Deviation.

2.03. Confidence Measures: Weight Loss and Weight Loss Maintenance (WLM).

Notes on scale and scale construction: Items for the perceived behavioral control (PBC), self-efficacy (SE) and confidence measures were adapted from previous scales [2,3]. Exploratory Factor Analyses (EFA) along with an examination of correlation matrices across the first two time points were conducted on these items to examine how they should be partitioned into scales. From these analyses, we determined that the two confidence items could be combined into a single factor, but that the average correlation between the two items was not as high as for other items (e.g., $r < .7$). Ultimately, however, we determined that it was theoretically beneficial to consider the domains of confidence towards weight loss, and confidence towards WLM, separately. Consequently, the items were not combined, but kept separate (see Table S9 vs. Table S10). Table S11 provides descriptive statistics for these variables.

Table S9*. Item Used to Measure Confidence: Weight Loss (1 item)*

**Items**	**Anchors**
How confident are you in your ability to... lose weight?	1= Not confident; 7 = confident

Table S10*. Item Used to Measure Confidence: Weight Loss Maintenance (WLM; 1 item)*

**Items**	**Anchors**
How confident are you in your ability to... maintain weight loss?	1= Not confident; 7 = confident

Table S11. *Descriptive Statistics per Time Point for PBC Variables.*

Time Point	Descriptive Statistic							
	**N**	**Mean**	**SD**	**Median**	**Min**	**Max**	**Skew**	**Kurtosis**
**Confidence: Weight Loss**								
Time 1	286	5.33	1.32	5.00	2.00	7.00	-.47	-.43
Time 2	231	4.66	1.70	5.00	1.00	7.00	-.48	-.61
Time 3	253	4.64	1.70	5.00	1.00	7.00	-.41	-.71
**Confidence: Weight Loss Maintenance**								
Time 1	286	4.23	1.55	4.00	1.00	7.00	-.21	-.55
Time 2	231	4.64	1.76	5.00	1.00	7.00	-.43	-.73
Time 3	253	4.66	1.78	5.00	1.00	7.00	-.40	-.82

Notes. N = number of responses per time point; SD = Standard Deviation.

2.04. Self-Efficacy (SE): Emotional Eating, Unhealthy Food Context, Physical Activity Barriers.

Notes on scale and scale construction: Items for the perceived behavioral control (PBC), self-efficacy (SE) and confidence measures were adapted from previous scales [2,3]. Exploratory Factor Analyses (EFA) along with an examination of correlation matrices across the first two time points were conducted on these items to examine how they should be partitioned into scales. From these analyses, we determined that SE items should be separated into three scales depending on whether they focused on: emotional eating, unhealthy food contexts, or physical activity barriers (i.e., Tables S12, S13 and S14). The key decision we made was to separate the first two factors from each other, but not have a similar division when it came to physical activity items (despite there being similar items tapping into being sedentary as the results of negative affect). This decision was made as EFAs showed a clear clustering of the emotional eating items (in Time 1-2, average inter-item around r = .80). This was much higher than their average correlation to the unhealthy food context items (r < .40), which, in turn tended to correlate with each other slightly more strongly (inter-item correlations for unhealthy food context items was around r = .50). In contrast, the items on physical activity barriers that dealt with negative affect and could feasibly be considered a separate cluster (i.e., the first 3 items in

Table S14, which correlated very highly with each other) did not deviate so strongly from the other physical activity barriers items (i.e., correlated with them on average r > .60). From a theoretical standpoint, emotional eating is often explicitly identified as a barrier to healthy eating, whereas the role of negative affect in reducing physical activity, although notable, is less prominent. Following this decision, we conducted CFAs using Time 3 data to confirm this factor structure. Table S15 presents factor loadings for CFA models across the time points. Table S16 provides descriptive statistics.

Table S12. *Items used to Measure Self-Efficacy - Emotional Eating (3 items)*

**Items**	**Anchors**
I can resist eating unhealthy food… when I am anxious	1= True; 4 = False
I can resist eating unhealthy food… when I am sad	1= True; 4 = False
I can resist eating unhealthy food… when I have experienced failure	1= True; 4 = False

Table S13. *Items used to Measure Self-Efficacy - Unhealthy Food Context (10 items)*

**Items**	**Anchors**
I can resist eating unhealthy food… when there are many different kinds of food available	1= True; 4 = False
I can resist eating unhealthy food… even when I am at a party	1= True; 4 = False
I can resist eating unhealthy food… even when high-calorie foods are available	1= True; 4 = False
I can resist eating unhealthy food… even when I have to say "no" to others	1= True; 4 = False
I can resist eating unhealthy food… even when I feel it's impolite to refuse a second helping	1= True; 4 = False
I can resist eating unhealthy food… even when others are pressuring me to eat	1= True; 4 = False
I can resist eating unhealthy food… when I am watching TV	1= True; 4 = False
I can resist eating unhealthy food… when I am reading	1= True; 4 = False
I can resist eating unhealthy food… just before going to bed	1= True; 4 = False
I can resist eating unhealthy food… even at weekends	1= True; 4 = False

Table S14. *Items used to Measure Self-Efficacy - Physical Activity Barriers (12 items)*

Items	Anchors
I will be physically active every day … … even when I have worries and problems	1= True; 4 = False
I will be physically active every day … … even when I am sad	1= True; 4 = False
I will be physically active every day … … even when I have experienced failure	1= True; 4 = False
I will be physically active every day … … even when I am tired	1= True; 4 = False
I will be physically active every day … … even when I am busy	1= True; 4 = False
I will be physically active every day … … even when the weather is cold or wet	1= True; 4 = False
I will be physically active every day … … even when others are pressuring me not to be physically active	1= True; 4 = False
I will be physically active every day … … even when I have things I'd prefer to do	1= True; 4 = False
I will be physically active every day … … even when I feel self-conscious	1= True; 4 = False
I will be physically active every day … … even when I have no-one to accompany me	1= True; 4 = False
I will be physically active every day … … even at weekends	1= True; 4 = False
I will be physically active every day … … even when I haven't been active for several days	1= True; 4 = False

Table S15. *Confirmatory Factor Analytic & Standardized Reliability Alphas for the Self-Efficacy Measures.*

Items	Standardized Factor Loadings[1]		
	T1	T2	T3
**SE: Emotional Eating**			
I can resist eating unhealthy food… when I am anxious	.86	.88	.90
I can resist eating unhealthy food… when I am sad	.92	.90	.93
I can resist eating unhealthy food… when I have experienced failure	.87	.91	.95
**SE: Unhealthy Food Context**			
I can resist eating unhealthy food… when there are many different kinds of food available	.73	.70	.80
I can resist eating unhealthy food… even when I am at a party	.71	.71	.78
I can resist eating unhealthy food… even when high-calorie foods are available	.79	.83	.86
I can resist eating unhealthy food… even when I have to say "no" to others	.80	.89	.88
I can resist eating unhealthy food… even when I feel it's impolite to refuse a second helping	.69	.84	.79

I can resist eating unhealthy food… even when others are pressuring me to eat	.72	.79	.77
I can resist eating unhealthy food… when I am watching TV	.52	.70	.62
I can resist eating unhealthy food… when I am reading	.41	.58	.62
I can resist eating unhealthy food… just before going to bed	.44	.54	.60
I can resist eating unhealthy food… even at weekends	.55	.66	.68
**SE: Physical Activity Barriers**			
I will be physically active every day… even when I have worries and problems	.88	.93	.90
I will be physically active every day … even when I am sad	.87	.94	.88
I will be physically active every day… even when I have experienced failure	.89	.94	.89
I will be physically active every day… even when I am tired	.80	.79	.82
I will be physically active every day… even when I am busy	.71	.73	.77
I will be physically active every day… even when the weather is cold or wet	.73	.76	.76
I will be physically active every day… even when others are pressuring me not to be physically active	.69	.79	.79
I will be physically active every day… even when I have things I'd prefer to do	.76	.78	.79
I will be physically active every day… even when I feel self-conscious	.76	.79	.78
I will be physically active every day… even when I have no-one to accompany me	.67	.72	.70
I will be physically active every day… even at weekends	.69	.69	.68
I will be physically active every day… even when I haven't been active for several days	.78	.79	.70
**Standardized Reliability Alphas**	T1	T2	T3
SE: Emotional eating	.91	.92	.95
SE: Unhealthy Food Context	.86	.92	.93
SE: Physical Activity Barriers	.95	.96	.95

[1]Loadings were calculated at each time point from a single CFA model that included all seven of the PBC, Confidence, and SE measures.

***Table S16**. Descriptive Statistics per Time Point for SE Variables.*

**Time Point**	**Descriptive Statistic**							
	**N**	**Mean**	**SD**	**Median**	**Min**	**Max**	**Skew**	**Kurtosis**
**SE: Emotional Eating**								
Time 1	287	2.60	0.91	2.67	1	4	-0.05	-0.94
Time 2	231	2.59	0.93	2.67	1	4	-0.04	-0.96
Time 3	253	2.48	0.95	2.33	1	4	0.07	-0.99
**SE: Unhealthy Food Context**								
Time 1	287	2.94	0.62	2.90	1	4	-0.47	0.08

Time 2	231	2.84	0.69	2.90	1	4	-0.33	-0.32
Time 3	253	2.87	0.72	2.90	1	4	-0.52	0.11
**SE: Physical Activity Barriers**								
Time 1	285	2.94	0.74	3.00	1	4	-0.56	-0.02
Time 2	230	2.75	0.8	2.75	1	4	-0.28	-0.59
Time 3	253	2.84	0.76	2.83	1	4	-0.41	-0.22

Notes. N = number of responses per time point; SD = Standard Deviation.

2.05. Action Planning & Coping Planning: Healthy Eating and Physical Activity.

Notes on scale and scale construction: Items for the action planning and coping planning measures were adapted from previous work [4]. Exploratory Factor Analyses (EFA) along with an examination of correlation matrices were conducted on these items using time points 1 to examine how they should be partitioned into scales. From these analyses, we determined that the items should be divided into four scales (i.e., Table S17, S18, S19, and S20). Inter-item correlations within scales were typically above *r* = .70, whereas the average inter-item correlation across scales was closer to *r* = .40. We then conducted CFAs using Time 2 and Time 3 data to confirm this factor structure. Table S21 presents factor loadings for CFA models across all time points (along with standardized reliability alphas). Table S22 provides descriptive statistics.

Table S17. *Items used to Measure Action Planning – Physical Activity (3 items)*

**Items**	**Anchors**
I have made a detailed plan regarding…when to be physically active	1=Totally agree; 4 = Totally disagree
I have made a detailed plan regarding…where to be physically active	1=Totally agree; 4 = Totally disagree
I have made a detailed plan regarding…how to be physically active	1=Totally agree; 4 = Totally disagree

Table S18. *Items used to Measure Action Planning – Healthy Eating (3 items)*

Items	Anchors
I have made a detailed plan regarding…when to make healthy food choices	1=Totally agree; 4 = Totally disagree
I have made a detailed plan regarding…where to make healthy food choices	1=Totally agree; 4 = Totally disagree
I have made a detailed plan regarding…what to do to make healthy food choices	1=Totally agree; 4 = Totally disagree

Table S19. *Items used to Measure Coping Planning – Physical Activity (3 items)*

Items	Anchors
I have made a detailed plan regarding…what to do if something interferes with my plans to be physically active	1=Totally agree; 4 = Totally disagree
I have made a detailed plan regarding…how to cope with possible setbacks when trying to be physically active	1=Totally agree; 4 = Totally disagree
I have made a detailed plan regarding…how to keep on being physically active in difficult situations	1=Totally agree; 4 = Totally disagree

Table S20. *Items used to Measure Coping Planning – Healthy Eating (4 items)*

Items	Anchors
I have made a detailed plan regarding…how to keep on eating healthily even when I am stressed	1=Totally agree; 4 = Totally disagree
I have made a detailed plan regarding…how to keep on eating healthily even when I am busy	1=Totally agree; 4 = Totally disagree
I have made a detailed plan regarding… what to do if I'm tempted by unhealthy foods	1=Totally agree; 4 = Totally disagree
I have made a detailed plan regarding… how to cope with friends and family pressuring me to eat unhealthily	1=Totally agree; 4 = Totally disagree

Table S21. *Confirmatory Factor Analytic & Standardized Reliability Alphas for Action Planning and Coping Planning Measures.*

Items	Standardized Factor Loadings		
	T1	T2	T3
**Action Planning – Physical Activity**			
I have made a detailed plan regarding…when to be physically active	.90	.94	.94
I have made a detailed plan regarding…where to be physically active	.96	.95	.96
I have made a detailed plan regarding…how to be physically active	.95	.94	.90
**Action Planning – Healthy Eating**			
I have made a detailed plan regarding…when to make healthy food choices	.92	.96	.98
I have made a detailed plan regarding…where to make healthy food choices	.99	.96	.97

I have made a detailed plan regarding…what to do to make healthy food choices	.92	.92	.92
**Coping Planning – Physical Activity**			
I have made a detailed plan regarding…what to do if something interferes with my plans to be physically active	.89	.91	.93
I have made a detailed plan regarding…how to cope with possible setbacks when trying to be physically active	.96	.97	.95
I have made a detailed plan regarding…how to keep on being physically active in difficult situations	.95	.95	.96
**Coping Planning – Healthy Eating**			
I have made a detailed plan regarding…how to keep on eating healthily even when I am stressed	.88	.90	.89
I have made a detailed plan regarding…how to keep on eating healthily even when I am busy	.85	.91	.90
I have made a detailed plan regarding… what to do if I'm tempted by unhealthy foods	.85	.90	.90
I have made a detailed plan regarding… how to cope with friends and family pressuring me to eat unhealthily	.83	.91	.88
**Standardized Reliability Alphas**	T1	T2	T3
Action Planning – Physical Activity	.95	.96	.95
Action Planning – Healthy Eating	.96	.96	.97
Coping Planning – Physical Activity	.95	.96	.96
Coping Planning – Healthy Eating	.91	.95	.94

Table S22. *Descriptive Statistics per Time Point for SE Variables.*

Time Point	Descriptive Statistic							
	**N**	**Mean**	**SD**	**Median**	**Min**	**Max**	**Skew**	**Kurtosis**
**Action Planning – Physical Activity**								
Time 1	287	2.87	1.03	3.00	1	4	-0.66	-0.80
Time 2	232	2.62	1.10	3.00	1	4	-0.30	-1.30
Time 3	253	2.76	1.06	3.00	1	4	-0.42	-1.11
**Action Planning – Healthy Eating**								
Time 1	287	3.22	0.79	3.00	1	4	-1.02	0.77
Time 2	232	2.99	0.92	3.00	1	4	-0.80	-0.13
Time 3	253	3.01	0.97	3.00	1	4	-0.83	-0.35
**Coping Planning – Physical Activity**								
Time 1	287	2.20	0.97	2.00	1	4	0.29	-1.06
Time 2	232	2.17	1.00	2.00	1	4	0.30	-1.13
Time 3	253	2.27	1.02	2.00	1	4	0.25	-1.09
**Coping Planning – Healthy Eating**								
Time 1	287	2.72	0.89	2.75	1	4	-0.34	-0.74
Time 2	232	2.64	0.92	2.75	1	4	-0.29	-0.79
Time 3	253	2.62	0.94	2.75	1	4	-0.20	-0.94

Notes. N = number of responses per time point; SD = Standard Deviation.

2.06. Automaticity: Healthy Eating, Physical Activity, and Self-Weighing.

Notes on scale and scale construction: Items for the automaticity measures were adapted from the Self-Report Behavioural Automaticity Index [5,6]. Exploratory Factor Analyses (EFA) along with an examination of correlation matrices were conducted on these items using time point 1 to examine how they should be partitioned into scales. From these analyses, we determined that the items should be divided into three scales (i.e., Table S23, S24, S25). Inter-item correlations within scales were on average close to r = .80, whereas the average inter-item correlation between scales was under r = .20. We then conducted CFAs using Time 2 and Time 3 data to confirm this factor structure. Table S26 presents factor loadings for CFA models across all time points (along with standardized reliability alphas). Table S27 provides descriptive statistics.

***Table S23**. Items used to Measure Automaticity: Healthy Eating (5 items)*

Items	Anchors
Making healthy food choices is something… I do automatically	1 = True; 4 = False
Making healthy food choices is something… I do without having to consciously remember	1 = True; 4 = False
Making healthy food choices is something… I do without thinking	1 = True; 4 = False
Making healthy food choices is something… I start doing before I realise I'm doing it	1 = True; 4 = False
Making healthy food choices is something… That's typically "me."	1 = True; 4 = False

***Table S24**. Items used to Measure Automaticity: Physical Activity (5 items)*

Items	Anchors
Being physically active is something… I do automatically	1 = True; 4 = False
Being physically active is something… I do without having to consciously remember	1 = True; 4 = False
Being physically active is something… I do without thinking	1 = True; 4 = False
Being physically active is something… I start doing before I realise I'm doing it	1 = True; 4 = False
Being physically active is something… That's typically "me."	1 = True; 4 = False

Table S25. *Items used to Measure Automaticity: Self-Weighing (5 items)*

**Items**	**Anchors**
Weighing myself is something… I do automatically	1 = True; 4 = False
Weighing myself is something… I do without having to consciously remember	1 = True; 4 = False
Weighing myself is something… I do without thinking	1 = True; 4 = False
Weighing myself is something… I start doing before I realise I'm doing it	1 = True; 4 = False
Weighing myself is something… That's typically "me."	1 = True; 4 = False

Table S26. *Confirmatory Factor Analytic & Standardized Reliability Alphas for Automaticity Measures.*

**Items**	**Standardized Factor Loadings**		
	**T1**	**T2**	**T3**
**Automaticity: Healthy Eating**			
Making healthy food choices is something… I do automatically	.82	.89	.85
Making healthy food choices is something… I do without having to consciously remember	.89	.93	.90
Making healthy food choices is something… I do without thinking	.91	.93	.96
Making healthy food choices is something… I start doing before I realise I'm doing it	.79	.89	.93
Making healthy food choices is something… That's typically "me."	.83	.83	.86
**Automaticity: Physical Activity**			
Being physically active is something… I do automatically	.94	.95	.94
Being physically active is something… I do without having to consciously remember	.95	.97	.96
Being physically active is something… I do without thinking	.95	.97	.98
Being physically active is something… I start doing before I realise I'm doing it	.86	.91	.93
Being physically active is something… That's typically "me."	.87	.89	.89
**Automaticity: Self-Weighing**			
Weighing myself is something… I do automatically	.84	.93	.94
Weighing myself is something… I do without having to consciously remember	.95	.96	.96
Weighing myself is something… I do without thinking	.95	.97	.98
Weighing myself is something… I start doing before I realise I'm doing it	.85	.88	.87
Weighing myself is something… That's typically "me."	.87	.88	.89
**Standardized Reliability Alphas**	T1	T2	T3
Automaticity: Healthy Eating	.93	.95	.95

Automaticity: Physical Activity	.96	.97	.97
Automaticity: Self-Weighing	.95	.97	.97

Table S27*. Descriptive Statistics per Time Point for Automaticity Variables.*

Time Point	Descriptive Statistic							
	**N**	**Mean**	**SD**	**Median**	**Min**	**Max**	**Skew**	**Kurtosis**
**Automaticity: Healthy Eating**								
Time 1	288	2.67	0.81	2.8	1	4	-0.04	-0.73
Time 2	231	2.78	0.82	2.8	1	4	-0.17	-0.80
Time 3	253	2.82	0.84	3.0	1	4	-0.11	-0.92
**Automaticity: Physical Activity**								
Time 1	288	2.39	0.89	2.2	1	4	0.22	-0.77
Time 2	231	2.43	0.98	2.4	1	4	0.10	-1.08
Time 3	253	2.54	0.97	2.4	1	4	0.07	-1.09
**Automaticity: Self-Weighing**								
Time 1	288	2.7	1.03	2.8	1	4	-0.20	-1.22
Time 2	231	2.93	0.98	3.0	1	4	-0.53	-0.87
Time 3	253	2.87	1.00	3.0	1	4	-0.39	-1.04

Notes. N = number of responses per time point; SD = Standard Deviation.

2.07. Energy and Drive.

Notes on scale and scale construction: Items for the energy and drive measure were developed for the current study. An examination of correlation matrices was conducted on these items using time points 1 to examine whether they should be partitioned into scales. From these analyses, we determined that the items should treated as a single cluster (as shown in Table S28). Theoretically, the scale could be divided into positive items (e.g., focusing on positive affective states) and negative items (e.g., focusing on negative affective states). However, this division did not seem warranted from an empirical viewpoint. The average correlation within the negative item category was r = .59; the average correlation within the positive item category was r = .52, and; the average correlation between the negative items and positive items categories was r = .48. We then conducted CFAs using Time 2 and Time 3 data to confirm this factor structure. Table S29 presents factor loadings for CFA models across all time points (along with

standardized reliability alphas). Table S29 provides descriptive statistics.

Table S28. *Items used to Measure Energy and Drive (12 items)*

Items	Anchors
Over the last 2 weeks… I have felt tired (R)	1=None of the time; 5 All of the time
Over the last 2 weeks… I have felt mentally exhausted (R)	1=None of the time; 5 All of the time
Over the last 2 weeks… I have felt full of energy	1 = All of the time; 5 = None of the Time
Over the last 2 weeks… I have felt drained (R)	1=None of the time; 5 All of the time
Over the last 2 weeks… I have found it easy to concentrate	1 = All of the time; 5 = None of the Time
Over the last 2 weeks… I have felt stressed (R)	1=None of the time; 5 All of the time
Over the last 2 weeks… I have felt alert and 'with it'	1 = All of the time; 5 = None of the Time
Over the last 2 weeks… I have felt full of vitality	1 = All of the time; 5 = None of the Time
Over the last 2 weeks… I have felt like I'm running on empty (R)	1=None of the time; 5 All of the time
Over the last 2 weeks… I have found it hard to keep trying with tasks (R)	1=None of the time; 5 All of the time
Over the last 2 weeks… I have easily solved problems	1 = All of the time; 5 = None of the Time
Over the last 2 weeks… I have felt distracted (R)	1=None of the time; 5 All of the time

(R) = Reverse-Coded Item

Table S29. *Confirmatory Factor Analytic & Standardized Reliability Alphas for Energy and Drive.*

Items	Standardized Factor Loadings		
	T1	T2	T3
Over the last 2 weeks… I have felt tired (R)	.77	.77	.83
Over the last 2 weeks… I have felt mentally exhausted (R)	.80	.81	.85
Over the last 2 weeks… I have felt full of energy	.77	.73	.88
Over the last 2 weeks… I have felt drained (R)	.70	.67	.70
Over the last 2 weeks… I have found it easy to concentrate	.75	.81	.84
Over the last 2 weeks… I have felt stressed (R)	.74	.77	.68
Over the last 2 weeks… I have felt alert and 'with it'	.75	.70	.69
Over the last 2 weeks… I have felt full of vitality	.69	.79	.71

Over the last 2 weeks… I have felt like I'm running on empty (R)	.65	.67	.73
Over the last 2 weeks… I have found it hard to keep trying with tasks (R)	.72	.77	.73
Over the last 2 weeks… I have easily solved problems	.72	.76	.74
Over the last 2 weeks… I have felt distracted (R)	.59	.56	.53
**Standardized Reliability Alphas**	.93	.93	.94

Table S30. Descriptive Statistics per Time Point for Energy and Drive.

**Time Point**	**Descriptive Statistic**							
	**N**	**Mean**	**SD**	**Median**	**Min**	**Max**	**Skew**	**Kurtosis**
**Energy and Drive**								
Time 1	287	3.45	0.72	3.50	1.08	5.00	-0.51	-0.04
Time 2	231	3.20	0.77	3.25	1.00	4.92	-0.17	-0.56
Time 3	253	3.24	0.81	3.33	1.08	4.92	-0.26	-0.74

Notes. N = number of responses per time point; SD = Standard Deviation.

3. Weight Assessment (Measurement Info, Correlations, and Descriptive Statistics)

At Time 1 and Time 3 weight (clothed, without shoes) was measured using a digital portable scale (SECA model 875; SECA UK Ltd, Birmingham, UK) to the nearest 0.1 kilogram (kg). Measurements were taken by research staff that were blind to participants' group allocation.

At Time 2 and Time 3, participants were also asked to self-report their weight. This was done by first asking participants what unit they preferred to express their weight in using the following question.

In the next question we are going to ask you to tell us your current weight. How would you prefer to report your weight?

- ❍ *Kilograms*
- ❍ *Pounds*
- ❍ *Stones and pounds*

Following this, participants reported their weight using their preferred metric using the following questions:

What is your current weight in kilograms? _________
What is your current weight in pounds? _________
What is your current weight in stones and pounds?

Stones _________
Pounds _________

Answers were converted to kg prior to analyses. Table S31 provides a correlation matrix of the weight measurements at all three time points. Although we only have self-report weight at Time 2, the availability of both the objective and self-report measures at Time 3 allow us to evaluate how comparable these assessment methods are. Specifically, self-report and objectively measured weight at Time 3 correlate at $r = .998$. This near-perfect correlation affords a certain

degree of confidence that self-report weight at Time 2 can be used in our path analysis models as a stand-in for objectively assessed weight at Time 2. For Time 1 and Time 3, our modelling can use the objective assessment of weight.

To give greater context for our results, Table S32 provides descriptive statistics on the objectively measured and self-reported weight variables across the three time points. Because only 68 participants provided both self-report and objective data on weight at Time 3, the lower portion of Table S32 provides additional info on only these 68 participants to compare their scores across both measurement methods. Overall, 41 (60%) of the 68 participants self-reported weights that were identical to the objectively measured weight, and only 8 (11%) self-reported a weight that differed by ≥ 1kg from their objectively measured weight (of these, only 3 differed by ≥ 2kg).

Table S31. *Correlation Table for Weight (Self-Report and Objectively Measured) Across Time Points.*

Time (Type of Assessment)	1.	2.	3.	4.
1. Time 1 (Objective)	-			
2. Time 2 (Subjective)	.884	-		
3. Time 3 (Objective)	.899	.895	-	
4. Time 3 (Subjective)	.888	.881	.998	-

Table S32. *Descriptive Statistics per Time Point for Weight (in Kg).*

Time Point	Descriptive Statistic							
	N	Mean	SD	Median	Min	Max	Skew	Kurtosis
**Considering all Responses**								
Time 1 (Objective)	288	85.54	16.70	83.70	50.00	163.80	0.89	1.42
Time 2 (Subjective)	221	84.28	17.91	80.45	48.53	180.00	1.26	3.55
Time 3 (Objective)	253	86.85	17.93	84.00	34.00	152.40	0.67	0.70
Time 3 (Subjective)	68	91.64	17.48	91.10	56.00	152.40	0.60	0.95
**Considering N = 68 who Gave Self-Reports and Objective Measurements at Time 3**								
Time 3 (Objective)	68	91.80	17.42	91.1	56.00	152.40	0.60	0.96
Time 3 Difference: Subjective – Objective	68	-0.16	1.00	0.00	-4.00	6.00	2.35	21.86

Notes. N = number of responses per time point; SD = Standard Deviation.

4. Detailed Results from Path Analyses

The complete path analytic model we computed for each psychological process variable is shown in Figure S1. Figure S1 labels each pathway in the model to ease the reporting of our findings. Our main text provides a summary of our findings for the primary paths of interest within the model; that is, *Path a*, *Path b*, and *Path a*b*. In this section of the supplemental materials, we provide results for each the full set of labelled paths in Figure S1. (a, b, c, d, e, f, g, h, I; these are provided in Tables S33 to S41) We also provide results for a few additional composite paths (including a*b, along with others, in Table S42 to S47). For each, we provide (1) a description of how to interpret the path, (2) the unstandardized regression coefficient corresponding to the path, (3) the 95% confidence interval for the unstandardized coefficient, (4) the standardized regression coefficient for the path, (5) the p-value associated with the path, and (6) the significance level of the test (e.g., <.05, <.01). Table S48 presents fit indices for each of the 16 path analytic models we conducted (R^2; CFAs, SRMR, RMSEA).

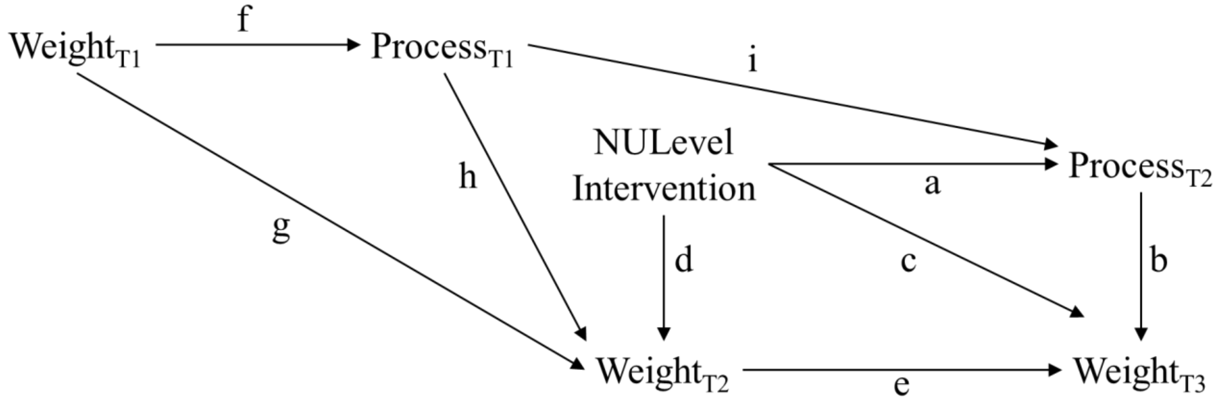

Figure S1. Path analytic model depicting our analyses.

Variable subscripts denote time points: T1 = Time 1; T2 = Time 2; T3 = Time 3.

4.01. Results: Path a

Path a captures the direct effect of the intervention on psychological processes at 6 months (T2). The results of the path analyses evaluating this effect are presented in Table S33. A *positive* number indicates that the intervention increased the psychological process at 6 months. This path was significant for 10 of 16 models.

Table S33. *Results Evaluating Path a of Figure S1.*

Model / Process	Path a					
	**B**	**SE**	**95% CI**	***β***	***p***	**sig.**
**01. Satisfaction with Changes**	.240	.079	[.083, .393]	.180	.002	**
**02. PBC: Healthy Eating**	.578	.152	[.284, .876]	.209	.000	***
**03. PBC: Physical Activity**	.208	.180	[-.143, .563]	.065	.247	
**04. Confidence: Weight Loss**	.534	.191	[.154, .898]	.158	.005	**
**05. Confidence: WLM**	1.062	.194	[.689, 1.451]	.305	.000	***
**06. SE: Emotional Eating**	-.075	.107	[-.282, .135]	-.040	.481	
**07. SE: Unhealthy Food Context**	.209	.077	[.058, .364]	.151	.007	**
**08. SE: Physical Activity Barriers**	.073	.085	[-.090, .244]	.045	.395	
**09. Action Planning: Physical Activity**	.153	.132	[-.102, .418]	.069	.248	
**10. Action Planning: Healthy Eating**	.365	.107	[.161, .576]	.200	.001	***
**11. Coping Planning: Physical Activity**	.228	.117	[.001, .465]	.114	.052	t
**12. Coping Planning: Healthy Eating**	.350	.107	[.145, .557]	.192	.001	**
**13. Automaticity: Healthy Eating**	.222	.090	[.050, .399]	.138	.013	*
**14. Automaticity: Physical Activity**	.090	.096	[-.094, .278]	.047	.345	
**15. Automaticity: Self-Weighing**	.491	.117	[.264, .717]	.252	.000	***
**16. Energy and Drive**	.032	.080	[-.123, .192]	.021	.690	

Notes. B = Unstandardized coefficient; SE = Standard Error; 95% CI = 95% Confidence Interval; β = Standardized Coefficient; p = p-value; sig. = significance level; PBC = Perceived Behavioral Control; WLM = Weight Loss Maintenance; SE = Self-Efficacy.
*** < .001; ** < .01; * < .05; ^{t} < .10

4.02. Results: Path b

Path b captures the direct effect of the psychological process (measured at 6 months) on weight at 12 months (T3). The results of the path analyses evaluating this effect are presented in Table S34. A *negative* number indicates that the psychological process at 6 months is associated with lower weight at 12 months. This path was significant for 3 of 16 models.

Table S34. *Results Evaluating Path b of Figure S1.*

**Model / Process**	**Path b**					
	**B**	**SE**	**95% CI**	***β***	***p***	**sig.**
**01. Satisfaction with Changes**	-.553	1.243	[-3.172, 1.662]	-.021	.657	
**02. PBC: Healthy Eating**	-1.124	.595	[-2.462, -.111]	-.089	.059	t
**03. PBC: Physical Activity**	-.568	.285	[-1.260, -.107]	-.051	.046	*
**04. Confidence: Weight Loss**	-.442	.440	[-1.462, .290]	-.042	.315	
**05. Confidence: WLM**	-.397	.462	[-1.358, .458]	-.039	.390	
**06. SE: Emotional Eating**	-.169	.358	[-.942, .474]	-.009	.638	
**07. SE: Unhealthy Food Context**	-.587	.534	[-1.897, .283]	-.023	.272	
**08. SE: Physical Activity Barriers**	-1.143	.744	[-2.820, .118]	-.051	.124	
**09. Action Planning: Physical Activity**	-.023	.345	[-.823, .566]	-.001	.946	
**10. Action Planning: Healthy Eating**	-.383	.584	[-1.774, .566]	-.020	.512	
**11. Coping Planning: Physical Activity**	-.457	.429	[-1.467, .256]	-.026	.287	
**12. Coping Planning: Healthy Eating**	-.513	.503	[-1.719, .319]	-.026	.308	
**13. Automaticity: Healthy Eating**	-3.053	1.261	[-5.769, -.748]	-.141	.016	*
**14. Automaticity: Physical Activity**	-.900	.552	[-2.208, -.005]	-.049	.103	
**15. Automaticity: Self-Weighing**	-.272	.478	[-1.364, .557]	-.015	.570	
**16. Energy and Drive**	-1.511	.644	[-3.003, -.430]	-.065	.019	*

Notes. B = Unstandardized coefficient; SE = Standard Error; 95% CI = 95% Confidence Interval; β = Standardized Coefficient; p = p-value; sig. = significance level; PBC = Perceived Behavioral Control; WLM = Weight Loss Maintenance; SE = Self-Efficacy.
*** < .001; ** < .01; * < .05; ^{t} < .10

4.03. Results: Path c

Path c captures the direct effect of the intervention on weight at 12 months (T3; accounting for paths a*b and d*e). The results of the path analyses evaluating this effect are presented in Table S35. A *negative* number indicates the intervention is associated with a lower weight at Time 3. This path was significant for 0 of 16 models.

Table S35. *Results Evaluating Path c of Figure S1.*

**Model / Process**	**Path c**					
	**B**	**SE**	**95% CI**	***β***	***p***	**sig.**
**01. Satisfaction with Changes**	.601	1.075	[-1.309, 2.934]	.017	.576	
**02. PBC: Healthy Eating**	1.270	1.134	[-.664, 3.851]	.036	.263	
**03. PBC: Physical Activity**	.717	1.057	[-1.256, 2.903]	.020	.498	
**04. Confidence: Weight Loss**	.751	1.088	[-1.148, 3.123]	.021	.490	
**05. Confidence: WLM**	.928	1.134	[-.983, 3.501]	.026	.414	
**06. SE: Emotional Eating**	.470	1.058	[-1.559, 2.612]	.013	.657	
**07. SE: Unhealthy Food Context**	.584	1.052	[-1.391, 2.756]	.016	.579	
**08. SE: Physical Activity Barriers**	.697	1.037	[-1.210, 2.873]	.020	.502	
**09. Action Planning: Physical Activity**	.495	1.059	[-1.501, 2.666]	.014	.640	
**10. Action Planning: Healthy Eating**	.644	1.189	[-1.505, 3.186]	.018	.588	
**11. Coping Planning: Physical Activity**	.598	1.079	[-1.411, 2.838]	.017	.579	
**12. Coping Planning: Healthy Eating**	.667	1.090	[-1.320, 2.992]	.019	.541	
**13. Automaticity: Healthy Eating**	1.410	1.151	[-.529, 4.056]	.041	.221	
**14. Automaticity: Physical Activity**	.719	1.080	[-1.288, 2.969]	.020	.506	
**15. Automaticity: Self-Weighing**	.607	1.013	[-1.299, 2.639]	.017	.549	
**16. Energy and Drive**	.597	1.048	[-1.358, 2.768]	.017	.569	

Notes. B = Unstandardized coefficient; SE = Standard Error; 95% CI = 95% Confidence Interval; β = Standardized Coefficient; p = p-value; sig. = significance level; PBC = Perceived Behavioral Control; WLM = Weight Loss Maintenance; SE = Self-Efficacy.
*** < .001; ** < .01; * < .05; ^{t} < .10

4.04. Results: Path d

Path d captures the direct effect of the intervention on weight at 6 months (T2). The results of the path analyses evaluating this effect are presented in Table S36. A *negative* number indicates that the intervention is decreased weight at 6 months. This path was significant for 0 of 16 models.

Table S36. *Results Evaluating Path d of Figure S1.*

**Model / Process**	**Path d**					
	**B**	**SE**	**95% CI**	***β***	***p***	**sig.**
**01. Satisfaction with Changes**	-.226	1.056	[-2.277, 1.868]	-.006	.831	
**02. PBC: Healthy Eating**	-.508	1.120	[-2.728, 1.653]	-.014	.650	
**03. PBC: Physical Activity**	-.277	1.058	[-2.336, 1.795]	-.008	.793	
**04. Confidence: Weight Loss**	-.373	1.093	[-2.532, 1.761]	-.011	.733	
**05. Confidence: WLM**	-.238	1.071	[-2.313, 1.874]	-.007	.824	
**06. SE: Emotional Eating**	-.035	1.137	[-2.181, 2.259]	-.001	.976	
**07. SE: Unhealthy Food Context**	-.184	1.067	[-2.274, 1.914]	-.005	.863	
**08. SE: Physical Activity Barriers**	-.261	1.055	[-2.343, 1.787]	-.007	.805	
**09. Action Planning: Physical Activity**	-.224	1.053	[-2.300, 1.837]	-.006	.832	
**10. Action Planning: Healthy Eating**	-.151	1.087	[-2.260, 2.010]	-.004	.890	
**11. Coping Planning: Physical Activity**	-.181	1.070	[-2.274, 1.951]	-.005	.866	
**12. Coping Planning: Healthy Eating**	-.146	1.106	[-2.293, 2.048]	-.004	.895	
**13. Automaticity: Healthy Eating**	-.395	1.071	[-2.515, 1.696]	-.011	.713	
**14. Automaticity: Physical Activity**	-.300	1.060	[-2.397, 1.751]	-.009	.777	
**15. Automaticity: Self-Weighing**	-.170	1.070	[-2.259, 1.962]	-.005	.874	
**16. Energy and Drive**	-.236	1.084	[-2.373, 1.906]	-.007	.828	

Notes. B = Unstandardized coefficient; SE = Standard Error; 95% CI = 95% Confidence Interval; β = Standardized Coefficient; p = p-value; sig. = significance level; PBC = Perceived Behavioral Control; WLM = Weight Loss Maintenance; SE = Self-Efficacy.
*** < .001; ** < .01; * < .05; ^{t} < .10

4.05. Results: Path e

Path e captures the direct effect of weight at 6 months (T2) on weight at 12 months (T3). The results of the path analyses evaluating this effect are presented in Table S37. A *positive* number indicates that weight at 6 months is positively associated with greater weight at 12 months. This path was significant for 16 of 16 models.

Table S37. *Results Evaluating Path e of Figure S1.*

**Model / Process**	**Path e**					
	**B**	**SE**	**95% CI**	***β***	***p***	**sig.**
**01. Satisfaction with Changes**	.902	.090	[.713, 1.056]	.895	.000	***
**02. PBC: Healthy Eating**	.875	.093	[.682, 1.034]	.883	.000	***
**03. PBC: Physical Activity**	.901	.086	[.716, 1.044]	.893	.000	***
**04. Confidence: Weight Loss**	.897	.091	[.708, 1.053]	.893	.000	***
**05. Confidence: WLM**	.901	.087	[.716, 1.049]	.894	.000	***
**06. SE: Emotional Eating**	.905	.085	[.722, 1.045]	.896	.000	***
**07. SE: Unhealthy Food Context**	.901	.088	[.712, 1.047]	.894	.000	***
**08. SE: Physical Activity Barriers**	.899	.087	[.718, 1.047]	.893	.000	***
**09. Action Planning: Physical Activity**	.907	.085	[.725, 1.047]	.896	.000	***
**10. Action Planning: Healthy Eating**	.902	.087	[.714, 1.046]	.895	.000	***
**11. Coping Planning: Physical Activity**	.906	.085	[.723, 1.046]	.895	.000	***
**12. Coping Planning: Healthy Eating**	.899	.089	[.709, 1.047]	.894	.000	***
**13. Automaticity: Healthy Eating**	.858	.094	[.673, 1.027]	.872	.000	***
**14. Automaticity: Physical Activity**	.904	.085	[.723, 1.046]	.893	.000	***
**15. Automaticity: Self-Weighing**	.906	.086	[.722, 1.047]	.896	.000	***
**16. Energy and Drive**	.897	.086	[.714, 1.041]	.889	.000	***

Notes. B = Unstandardized coefficient; SE = Standard Error; 95% CI = 95% Confidence Interval; β = Standardized Coefficient; p = p-value; sig. = significance level; PBC = Perceived Behavioral Control; WLM = Weight Loss Maintenance; SE = Self-Efficacy.
*** < .001; ** < .01; * < .05; ^{t} < .10

4.06. Results: Path f

Path f captures the association between weight at baseline (T1) and the psychological process at baseline (T1). The results of the path analyses evaluating this effect are presented in Table S38. A *negative* number indicates that a higher weight at baseline is associated with a lower level of the psychological process at baseline. This path weas significant in 12 of 16 models.

Table S38*. Results Evaluating Path f of Figure S1.*

Model / Process	Path f					
	**B**	**SE**	**95% CI**	***β***	***p***	**sig.**
**01. Satisfaction with Changes**	-.007	.002	[-.010, -.004]	-.217	.000	***
**02. PBC: Healthy Eating**	-.021	.004	[-.029, -.014]	-.297	.000	***
**03. PBC: Physical Activity**	-.014	.005	[-.024, -.004]	-.153	.006	**
**04. Confidence: Weight Loss**	-.013	.005	[-.022, -.004]	-.161	.006	**
**05. Confidence: WLM**	-.011	.005	[-.022, -.001]	-.121	.035	*
**06. SE: Emotional Eating**	-.006	.003	[-.012, .000]	-.107	.069	t
**07. SE: Unhealthy Food Context**	-.008	.002	[-.012, -.004]	-.212	.000	***
**08. SE: Physical Activity Barriers**	-.006	.003	[-.012, -.001]	-.141	.020	*
**09. Action Planning: Physical Activity**	.004	.004	[-.004, .011]	.059	.338	
**10. Action Planning: Healthy Eating**	-.004	.003	[-.009, .001]	-.087	.119	
**11. Coping Planning: Physical Activity**	-.004	.003	[-.011, .002]	-.074	.213	
**12. Coping Planning: Healthy Eating**	-.009	.003	[-.015, -.003]	-.162	.006	**
**13. Automaticity: Healthy Eating**	-.015	.003	[-.021, -.010]	-.313	.000	***
**14. Automaticity: Physical Activity**	-.008	.003	[-.015, -.002]	-.154	.008	**
**15. Automaticity: Self-Weighing**	-.007	.003	[-.014, -.000]	-.114	.043	*
**16. Energy and Drive**	-.010	.002	[-.015, -.005]	-.228	.000	***

Notes. B = Unstandardized coefficient; SE = Standard Error; 95% CI = 95% Confidence Interval; β = Standardized Coefficient; p = p-value; sig. = significance level; PBC = Perceived Behavioral Control; WLM = Weight Loss Maintenance; SE = Self-Efficacy.
*** < .001; ** < .01; * < .05; ^{t} < .10

4.07. Results: Path g

Path g captures the direct effect of weight at baseline (T1) on weight at 6 months (T2). The results of the path analyses evaluating this effect are presented in Table S39. A positive number indicates that higher baseline weight is associated with higher weight at 6 months. The path for significant for all 16 of 16 models.

Table S39. *Results Evaluating Path g of Figure S1.*

**Model / Process**	**Path g**					
	**B**	**SE**	**95% CI**	***β***	***p***	**sig.**
**01. Satisfaction with Changes**	.956	.047	[.886, 1.075]	.904	.000	***
**02. PBC: Healthy Eating**	.968	.047	[.896, 1.087]	.912	.000	***
**03. PBC: Physical Activity**	.948	.049	[.872, 1.065]	.896	.000	***
**04. Confidence: Weight Loss**	.948	.048	[.875, 1.069]	.896	.000	***
**05. Confidence: WLM**	.945	.047	[.872, 1.059]	.893	.000	***
**06. SE: Emotional Eating**	.934	.046	[.858, 1.040]	.884	.000	***
**07. SE: Unhealthy Food Context**	.935	.049	[.859, 1.054]	.884	.000	***
**08. SE: Physical Activity Barriers**	.944	.050	[.865, 1.067]	.892	.000	***
**09. Action Planning: Physical Activity**	.939	.049	[.861, 1.056]	.888	.000	***
**10. Action Planning: Healthy Eating**	.937	.047	[.861, 1.050]	.886	.000	***
**11. Coping Planning: Physical Activity**	.937	.047	[.860, 1.049]	.887	.000	***
**12. Coping Planning: Healthy Eating**	.936	.047	[.861, 1.048]	.885	.000	***
**13. Automaticity: Healthy Eating**	.954	.049	[.877, 1.075]	.900	.000	***
**14. Automaticity: Physical Activity**	.946	.050	[.868, 1.066]	.894	.000	***
**15. Automaticity: Self-Weighing**	.935	.046	[.857, 1.042]	.885	.000	***
**16. Energy and Drive**	.944	.047	[.871, 1.057]	.893	.000	***

Notes. B = Unstandardized coefficient; SE = Standard Error; 95% CI = 95% Confidence Interval; β = Standardized Coefficient; p = p-value; sig. = significance level; PBC = Perceived Behavioral Control; WLM = Weight Loss Maintenance; SE = Self-Efficacy.
*** < .001; ** < .01; * < .05; ^{t} < .10

4.08. Results: Path h

Path h captures the association between the psychological process at baseline (T1) and weight at 6 months (T2). The results of the path analyses evaluating this effect are presented in Table S40. A negative number indicates that a higher level of the process variable is associated with a lower 6-month weight. This path was significant for only 1 of 16 models.

Table S40. *Results Evaluating Path h of Figure S1.*

**Model / Process**	**Path h**					
	**B**	**SE**	**95% CI**	***β***	***p***	**sig.**
**01. Satisfaction with Changes**	2.809	1.463	[.338, 6.112]	.081	.055	t
**02. PBC: Healthy Eating**	1.234	.605	[.201, 2.577]	.083	.041	*
**03. PBC: Physical Activity**	.714	.383	[.029, 1.525]	.061	.062	t
**04. Confidence: Weight Loss**	.797	.521	[-.090, 1.970]	.059	.126	
**05. Confidence: WLM**	.565	.438	[-.176, 1.548]	.050	.197	
**06. SE: Emotional Eating**	-.552	.701	[-2.127, .659]	-.028	.431	
**07. SE: Unhealthy Food Context**	-.355	.767	[-1.944, 1.099]	-.012	.644	
**08. SE: Physical Activity Barriers**	.901	.725	[-.351, 2.510]	.038	.214	
**09. Action Planning: Physical Activity**	-.349	.527	[-1.603, .508]	-.020	.508	
**10. Action Planning: Healthy Eating**	-.183	.529	[-1.272, .815]	-.008	.729	
**11. Coping Planning: Physical Activity**	.003	.490	[-.982, .942]	.000	.994	
**12. Coping Planning: Healthy Eating**	-.180	.551	[-1.264, .905]	-.009	.743	
**13. Automaticity: Healthy Eating**	.962	.698	[-.327, 2.383]	.044	.168	
**14. Automaticity: Physical Activity**	.926	.572	[-.128, 2.123]	.047	.106	
**15. Automaticity: Self-Weighing**	-.322	.546	[-1.548, .623]	-.019	.555	
**16. Energy and Drive**	.700	.824	[-.861, 2.394]	.029	.395	

Notes. B = Unstandardized coefficient; SE = Standard Error; 95% CI = 95% Confidence Interval; β = Standardized Coefficient; p = p-value; sig. = significance level; PBC = Perceived Behavioral Control; WLM = Weight Loss Maintenance; SE = Self-Efficacy.
*** < .001; ** < .01; * < .05; ^{t} < .10

4.09. Results: Path i

Path i captures the direct association between the psychological processes at baseline (T1) and the psychological processes at 6 months (T2). The results of the path analyses evaluating this effect are presented in Table S41. A positive number indicates stability such that higher levels of the processes at baseline predict higher levels of the processes at 6 months. This path was significant for all 16 of 16 models.

Table S41. *Results Evaluating Path i of Figure S1.*

**Model / Process**	**Path i**					
	**B**	**SE**	**95% CI**	***β***	***p***	**sig.**
**01. Satisfaction with Changes**	.569	.075	[.418, .716]	.433	.000	***
**02. PBC: Healthy Eating**	.619	.066	[.495, .753]	.530	.000	***
**03. PBC: Physical Activity**	.559	.060	[.440, .673]	.524	.000	***
**04. Confidence: Weight Loss**	.655	.074	[.503, .791]	.511	.000	***
**05. Confidence: WLM**	.507	.060	[.383, .621]	.451	.000	***
**06. SE: Emotional Eating**	.496	.063	[.366, .614]	.484	.000	***
**07. SE: Unhealthy Food Context**	.557	.069	[.420, .687]	.496	.000	***
**08. SE: Physical Activity Barriers**	.639	.063	[.508, .753]	.594	.000	***
**09. Action Planning: Physical Activity**	.445	.063	[.318, .564]	.416	.000	***
**10. Action Planning: Healthy Eating**	.476	.071	[.330, .610]	.412	.000	***
**11. Coping Planning: Physical Activity**	.449	.061	[.327, .565]	.439	.000	***
**12. Coping Planning: Healthy Eating**	.437	.060	[.316, .553]	.427	.000	***
**13. Automaticity: Healthy Eating**	.508	.059	[.389, .623]	.513	.000	***
**14. Automaticity: Physical Activity**	.720	.050	[.613, .813]	.662	.000	***
**15. Automaticity: Self-Weighing**	.335	.057	[.224, .447]	.354	.000	***
**16. Energy and Drive**	.646	.059	[.528, .756]	.612	.000	***

Notes. B = Unstandardized coefficient; SE = Standard Error; 95% CI = 95% Confidence Interval; β = Standardized Coefficient; p = p-value; sig. = significance level; PBC = Perceived Behavioral Control; WLM = Weight Loss Maintenance; SE = Self-Efficacy.
*** < .001; ** < .01; * < .05; ^{t} < .10

4.10. Results: Composite Path a*b

Path a*b captures the indirect effect of the intervention on weight at 12 months (T3) attributed to changes on the process at 6 months (T2; accounting for paths c and d*e). The results of the path analyses evaluating this effect are presented in Table S42. A negative number indicates an indirect benefit effect of the intervention (i.e., decreased weight) attributable to the intervention's effect on the psychological process at 6 months. This path was significant in 0 of 16 models.

***Table S42**. Results Evaluating Path a*b of Figure S1.*

Model / Process	Path a*b					
	**B**	**SE**	**95% CI**	***β***	***p***	**sig.**
**01. Satisfaction with Changes**	-.133	.312	[-.913, .396]	-.004	.670	
**02. PBC: Healthy Eating**	-.649	.388	[-1.741, -.093]	-.019	.094	t
**03. PBC: Physical Activity**	-.118	.126	[-.544, .030]	-.003	.349	
**04. Confidence: Weight Loss**	-.236	.261	[-1.030, .115]	-.007	.366	
**05. Confidence: WLM**	-.421	.499	[-1.534, .478]	-.012	.399	
**06. SE: Emotional Eating**	.013	.050	[-.038, .209]	.000	.799	
**07. SE: Unhealthy Food Context**	-.123	.127	[-.541, .037]	-.003	.335	
**08. SE: Physical Activity Barriers**	-.083	.125	[-.553, .040]	-.002	.507	
**09. Action Planning: Physical Activity**	-.004	.071	[-.220, .103]	.000	.960	
**10. Action Planning: Healthy Eating**	-.140	.230	[-.793, .192]	-.004	.544	
**11. Coping Planning: Physical Activity**	-.104	.125	[-.546, .032]	-.003	.404	
**12. Coping Planning: Healthy Eating**	-.179	.193	[-.768, .083]	-.005	.351	
**13. Automaticity: Healthy Eating**	-.678	.387	[-1.861, -.149]	-.020	.080	t
**14. Automaticity: Physical Activity**	-.081	.111	[-.482, .037]	-.002	.465	
**15. Automaticity: Self-Weighing**	-.133	.249	[-.794, .250]	-.004	.592	
**16. Energy and Drive**	-.048	.131	[-.403, .157]	-.001	.711	

Notes. B = Unstandardized coefficient; SE = Standard Error; 95% CI = 95% Confidence Interval; β = Standardized Coefficient; p = p-value; sig. = significance level; PBC = Perceived Behavioral Control; WLM = Weight Loss Maintenance; SE = Self-Efficacy.
*** < .001; ** < .01; * < .05; ^{t} < .10

4.11. Results: Composite Path d*e

Path d*e captures the indirect effect of the intervention on weight at 12 months (T3) attributed to changes on weight at 6 months (T2). The results of the path analyses evaluating this effect are presented in Table S43. A negative number indicates an indirect benefit effect of the intervention (i.e., decreased weight) attributable to the intervention's effect on weight at 6 months. This path was significant in 0 of 16 models.

Table S43. *Results Evaluating Path d*e of Figure S1.*

**Model / Process**	**Path d*e**					
	**B**	**SE**	**95% CI**	***β***	***p***	**sig.**
**01. Satisfaction with Changes**	-.204	.951	[-2.117, 1.596]	-.006	.830	
**02. PBC: Healthy Eating**	-.445	.983	[-2.452, 1.388]	-.013	.651	
**03. PBC: Physical Activity**	-.250	.951	[-2.166, 1.567]	-.007	.793	
**04. Confidence: Weight Loss**	-.334	.979	[-2.331, 1.522]	-.009	.733	
**05. Confidence: WLM**	-.214	.962	[-2.145, 1.614]	-.006	.824	
**06. SE: Emotional Eating**	-.031	1.021	[-2.062, 1.912]	-.001	.975	
**07. SE: Unhealthy Food Context**	-.166	.957	[-2.085, 1.635]	-.005	.862	
**08. SE: Physical Activity Barriers**	-.235	.949	[-2.149, 1.568]	-.007	.805	
**09. Action Planning: Physical Activity**	-.203	.952	[-2.124, 1.598]	-.006	.831	
**10. Action Planning: Healthy Eating**	-.136	.974	[-2.085, 1.711]	-.004	.889	
**11. Coping Planning: Physical Activity**	-.163	.965	[-2.104, 1.676]	-.005	.866	
**12. Coping Planning: Healthy Eating**	-.131	.989	[-2.113, 1.738]	-.004	.895	
**13. Automaticity: Healthy Eating**	-.338	.917	[-2.191, 1.390]	-.010	.712	
**14. Automaticity: Physical Activity**	-.271	.958	[-2.206, 1.525]	-.008	.777	
**15. Automaticity: Self-Weighing**	-.154	.966	[-2.085, 1.687]	-.004	.873	
**16. Energy and Drive**	-.212	.970	[-2.151, 1.637]	-.006	.827	

Notes. B = Unstandardized coefficient; SE = Standard Error; 95% CI = 95% Confidence Interval; β = Standardized Coefficient; p = p-value; sig. = significance level; PBC = Perceived Behavioral Control; WLM = Weight Loss Maintenance; SE = Self-Efficacy.
*** < .001; ** < .01; * < .05; ^{t} < .10

4.12. Results: Composite Path "decab" [(d*e) + c + (a*b)]

Path "decab" [(d*e) + c + (a*b)] captures the total effect of the intervention on weight at 12 months (sum of the effects of path a, path a*b, and path d*e). The results of the path analyses evaluating this effect are presented in Table S44. A positive number indicates that the intervention increased weight at 12 months (accounting both for direct and indirect effects). This path was significant in 0 of 16 models.

Table S44. *Results Evaluating Path "decab" [(d*e) + c + (a*b)] of Figure S1.*

**Model / Process**	**Path "decab" [(d*e) + c + (a*b)]**					
	**B**	**SE**	**95% CI**	***β***	***p***	**sig.**
**01. Satisfaction with Changes**	.264	.988	[-1.630, 2.224]	.007	.789	
**02. PBC: Healthy Eating**	.176	1.038	[-1.780, 2.257]	.005	.865	
**03. PBC: Physical Activity**	.349	1.005	[-1.572, 2.329]	.010	.729	
**04. Confidence: Weight Loss**	.181	1.010	[-1.758, 2.185]	.005	.858	
**05. Confidence: WLM**	.292	1.005	[-1.630, 2.282]	.008	.771	
**06. SE: Emotional Eating**	.451	1.025	[-1.524, 2.480]	.013	.660	
**07. SE: Unhealthy Food Context**	.296	1.005	[-1.654, 2.267]	.008	.769	
**08. SE: Physical Activity Barriers**	.379	1.011	[-1.560, 2.360]	.011	.708	
**09. Action Planning: Physical Activity**	.289	1.015	[-1.661, 2.272]	.008	.776	
**10. Action Planning: Healthy Eating**	.368	1.009	[-1.558, 2.389]	.010	.715	
**11. Coping Planning: Physical Activity**	.331	1.005	[-1.574, 2.324]	.009	.742	
**12. Coping Planning: Healthy Eating**	.356	1.010	[-1.578, 2.362]	.010	.724	
**13. Automaticity: Healthy Eating**	.393	1.027	[-1.556, 2.482]	.011	.702	
**14. Automaticity: Physical Activity**	.367	1.010	[-1.566, 2.370]	.010	.717	
**15. Automaticity: Self-Weighing**	.320	1.011	[-1.632, 2.313]	.009	.752	
**16. Energy and Drive**	.337	1.005	[-1.584, 2.320]	.009	.737	

Notes. B = Unstandardized coefficient; SE = Standard Error; 95% CI = 95% Confidence Interval; β = Standardized Coefficient; p = p-value; sig. = significance level; PBC = Perceived Behavioral Control; WLM = Weight Loss Maintenance; SE = Self-Efficacy.
*** < .001; ** < .01; * < .05; ^{t} < .10

4.13. Results: Composite Path "ibhe" [(i*b) + (h*e)]

Path "ibhe" [(i*b) + (h*e)] captures the total effect of psychological processes at baseline on weight at 12 months (i.e., sum of path i*b and path h*e). The results of the path analyses evaluating this effect are presented in Table S45. A negative number indicates the psychological process at 6 months predicted lower weight at 12 months. This path was significant in 0 of 16 models.

Table S45*. Results Evaluating Path "ibhe" [(i*b) + (h*e)] of Figure S1.*

**Model / Process**	**Path "ibhe" [(i*b) + (h*e)]**					
	**B**	**SE**	**95% CI**	***β***	***p***	**sig.**
**01. Satisfaction with Changes**	2.220	1.144	[.115, 4.641]	.064	.052	t
**02. PBC: Healthy Eating**	.384	.643	[-.879, 1.653]	.026	.550	
**03. PBC: Physical Activity**	.325	.340	[-.309, 1.014]	.027	.339	
**04. Confidence: Weight Loss**	.425	.393	[-.298, 1.268]	.032	.279	
**05. Confidence: WLM**	.308	.368	[-.395, 1.050]	.027	.403	
**06. SE: Emotional Eating**	-.583	.664	[-1.907, .690]	-.030	.379	
**07. SE: Unhealthy Food Context**	-.647	.773	[-2.243, .796]	-.022	.403	
**08. SE: Physical Activity Barriers**	.080	.592	[-1.038, 1.273]	.003	.892	
**09. Action Planning: Physical Activity**	-.327	.503	[-1.391, .572]	-.019	.516	
**10. Action Planning: Healthy Eating**	-.347	.523	[-1.387, .675]	-.015	.506	
**11. Coping Planning: Physical Activity**	-.202	.500	[-1.194, .769]	-.011	.686	
**12. Coping Planning: Healthy Eating**	-.386	.546	[-1.472, .665]	-.019	.479	
**13. Automaticity: Healthy Eating**	-.726	.786	[-2.351, .737]	-.034	.356	
**14. Automaticity: Physical Activity**	.189	.560	[-.951, 1.241]	.009	.735	
**15. Automaticity: Self-Weighing**	-.383	.529	[-1.467, .574]	-.022	.469	
**16. Energy and Drive**	-.348	.813	[-2.009, 1.165]	-.014	.669	

Notes. B = Unstandardized coefficient; SE = Standard Error; 95% CI = 95% Confidence Interval; β = Standardized Coefficient; p = p-value; sig. = significance level; PBC = Perceived Behavioral Control; WLM = Weight Loss Maintenance; SE = Self-Efficacy.
*** < .001; ** < .01; * < .05; ^{t} < .10

4.14. Results: Composite Path "gefhe" [(g*e + f*h*e)]

Path "gefhe" [(g*e + f*h*e)] captures the stability of weight that operates through weight at 6 months (i.e., sum of path g*e, and path f*h*e). The results of the path analyses evaluating this effect are presented in Table S46. A positive number indicates that weight at baseline positively predicted weight at 12 months through these paths. This path was significant in all 16 of 16 models.

Table S46. *Results Evaluating Path a"gefhe" [(g*e + f*h*e)] of Figure S1.*

**Model / Process**	**Path "gefhe" [(g*e + f*h*e)]**					
	**B**	**SE**	**95% CI**	***β***	***p***	**sig.**
**01. Satisfaction with Changes**	.846	.074	[.691, .978]	.793	.000	***
**02. PBC: Healthy Eating**	.824	.074	[.672, .958]	.783	.000	***
**03. PBC: Physical Activity**	.845	.070	[.697, .966]	.792	.000	***
**04. Confidence: Weight Loss**	.841	.073	[.690, .973]	.792	.000	***
**05. Confidence: WLM**	.846	.071	[.698, .971]	.794	.000	***
**06. SE: Emotional Eating**	.848	.068	[.706, .967]	.794	.000	***
**07. SE: Unhealthy Food Context**	.845	.070	[.696, .968]	.792	.000	***
**08. SE: Physical Activity Barriers**	.844	.070	[.698, .968]	.792	.000	***
**09. Action Planning: Physical Activity**	.850	.068	[.705, .970]	.795	.000	***
**10. Action Planning: Healthy Eating**	.846	.070	[.695, .969]	.794	.000	***
**11. Coping Planning: Physical Activity**	.849	.068	[.704, .969]	.794	.000	***
**12. Coping Planning: Healthy Eating**	.843	.071	[.693, .969]	.792	.000	***
**13. Automaticity: Healthy Eating**	.805	.078	[.648, .948]	.773	.000	***
**14. Automaticity: Physical Activity**	.849	.069	[.703, .969]	.793	.000	***
**15. Automaticity: Self-Weighing**	.849	.069	[.703, .970]	.794	.000	***
**16. Energy and Drive**	.841	.069	[.696, .964]	.788	.000	***

Notes. B = Unstandardized coefficient; SE = Standard Error; 95% CI = 95% Confidence Interval; β = Standardized Coefficient; p = p-value; sig. = significance level; PBC = Perceived Behavioral Control; WLM = Weight Loss Maintenance; SE = Self-Efficacy.
*** < .001; ** < .01; * < .05; ^{t} < .10

4.15. Results: Composite Path "gefhefib" [(g*e + f*h*e + f*i*b)]

Path "gefhefib" [(g*e + f*h*e + f*i*b)] captures the total stability effect of weight at baseline (T1) on weight at 12 months (T3) that operates through any indirect path (sum of path g*h, path f*h*e, and path f*i*b). The results of the path analyses evaluating this effect are presented in Table S47. A positive number indicates that weight at baseline positively predicted weight at 12 months through these paths. This path was significant in all 16 of 16 models.

Table S47*. Results Evaluating Path "gefhefib" [(g*e + f*h*e + f*i*b)] of Figure S1.*

**Model / Process**	**Path "gefhefib" [(g*e + f*h*e + f*h*e)]**					
	**B**	**SE**	**95% CI**	***β***	***p***	**sig.**
**01. Satisfaction with Changes**	.848	.070	[.701, .974]	.795	.000	***
**02. PBC: Healthy Eating**	.839	.069	[.696, .963]	.797	.000	***
**03. PBC: Physical Activity**	.849	.068	[.706, .968]	.796	.000	***
**04. Confidence: Weight Loss**	.845	.070	[.700, .972]	.795	.000	***
**05. Confidence: WLM**	.848	.069	[.704, .970]	.796	.000	***
**06. SE: Emotional Eating**	.849	.068	[.705, .968]	.795	.000	***
**07. SE: Unhealthy Food Context**	.847	.069	[.701, .969]	.795	.000	***
**08. SE: Physical Activity Barriers**	.848	.068	[.705, .968]	.796	.000	***
**09. Action Planning: Physical Activity**	.850	.068	[.706, .969]	.795	.000	***
**10. Action Planning: Healthy Eating**	.847	.070	[.698, .969]	.794	.000	***
**11. Coping Planning: Physical Activity**	.849	.068	[.705, .969]	.795	.000	***
**12. Coping Planning: Healthy Eating**	.845	.070	[.697, .969]	.794	.000	***
**13. Automaticity: Healthy Eating**	.829	.069	[.691, .957]	.796	.000	***
**14. Automaticity: Physical Activity**	.854	.066	[.714, .971]	.798	.000	***
**15. Automaticity: Self-Weighing**	.849	.068	[.705, .970]	.795	.000	***
**16. Energy and Drive**	.850	.066	[.711, .968]	.797	.000	***

Notes. B = Unstandardized coefficient; SE = Standard Error; 95% CI = 95% Confidence Interval; β = Standardized Coefficient; p = p-value; sig. = significance level; PBC = Perceived Behavioral Control; WLM = Weight Loss Maintenance; SE = Self-Efficacy.
*** < .001; ** < .01; * < .05; ^{t} < .10

4.16. Results: Fit Indices and R2 Values for Each Path Analytic Model.

Table S48 presents fit indices for each of the 16 path analytic models we conducted. The coefficient of determination (R^2) are provided for processes at baseline (T1), processes at 6 months (T2), weight at 6 months (T2) and weight at 12 months (T3).

***Table S48**. Fit Indices and R^2 Values for Each Path Analytic Model*

Model / Process	Fit Indices				R^2			
	CFI	RMSEA		SRMR	Process		Weight	
		Est.	95% CI		T1	T2	T2	T3
**01. Satisfaction with Changes**	.854	.281	[.242, .322]	.059	.047	.220	.792	.803
**02. PBC: Healthy Eating**	.881	.261	[.222, .302]	.077	.088	.324	.794	.803
**03. PBC: Physical Activity**	.902	.226	[.187, .267]	.036	.024	.279	.791	.804
**04. Confidence: Weight Loss**	.887	.246	[.207, .287]	.064	.026	.286	.789	.803
**05. Confidence: WLM**	.908	.219	[.180, .260]	.041	.015	.296	.790	.803
**06. SE: Emotional Eating**	.885	.244	[.205, .285]	.064	.011	.236	.787	.804
**07. SE: Unhealthy Food Context**	.894	.236	[.197, .278]	.051	.045	.269	.786	.803
**08. SE: Physical Activity Barriers**	.908	.222	[.183, .263]	.038	.020	.355	.788	.804
**09. Action Planning: Physical Activity**	.909	.212	[.173, .253]	.031	.004	.178	.787	.804
**10. Action Planning: Healthy Eating**	.898	.227	[.188, .268]	.065	.008	.209	.786	.803
**11. Coping Planning: Physical Activity**	.907	.215	[.176, .257]	.023	.006	.206	.786	.804
**12. Coping Planning: Healthy Eating**	.893	.235	[.196, .276]	.069	.026	.219	.786	.802
**13. Automaticity: Healthy Eating**	.893	.245	[.206, .286]	.076	.098	.282	.787	.811
**14. Automaticity: Physical Activity**	.920	.210	[.171, .251]	.022	.024	.441	.789	.806
**15. Automaticity: Self-Weighing**	.898	.227	[.188, .268]	.025	.013	.189	.787	.804
**16. Energy and Drive**	.918	.211	[.172, .252]	.030	.052	.376	.787	.807

PBC = Perceived Behavioral Control; WLM = Weight Loss Maintenance; SE = Self-Efficacy; CFI = Comparative Fit Index; RMSEA = Root Mean Square Error of Approximation; SRMR = Standardized Root Mean Square Residual; Est. = Estimate; CI = Confidence Interval; R^2 = Coefficient of Determination; T1 = Time 1; T2 = Time 2; T3 = Time 3.

5. Results: *T*-*T*ests and Descriptives of the Change in Each Process from Time 1 to Time 2

Table S49 provides a descriptive examination of how each process variable changed from Time 1 (T1) to Time 2 (T2), comparing between the control and intervention groups. Descriptive statistics are provided for each variable (i.e., means at T1 and T2), along with the results of paired samples t-tests (comparing T2 scores minus T1 scores). The t-test results provide the estimates change, 95% confidence intervals, the *t* statistic, and the corresponding *p* value. To facilitate a comparison across variables, each difference score has also been converted to a standardized metric (Cohen's *d*).

Table S49. *Descriptives and t-tests Examining changes in each Process Variable from T1 (baseline) to T2 (6 months).*

	Control								Intervention							
**Process Variable**	**Descriptive**		**Paired Samples t-test (T2-T1)**						**Descriptive**		**Paired Samples t-test (T2-T1)**					
	**Means (SD)**		**est.**	**95% *CI***		***t***	***p***	***d***	**Means (SD)**		**est.**	**95% *CI***		***t***	***p***	***d***
	**T2**	**T1**		**low**	**high**				**T2**	**T1**		**low**	**high**			
**01. Satisfaction with Changes**	2.22 (.69)	2.63 (.53)	-.43	-.55	-.30	-6.66	<.01	**-.62**	2.47 (.63)	2.65 (.49)	-.19	-.30	-.09	-3.63	<.01	**-.34**
**02. PBC: Healthy Eating**	4.82 (1.47)	5.10 (1.19)	-.33	-.57	-.10	-2.79	.01	**-.26**	5.60 (1.25)	5.41 (1.17)	.13	-.09	.35	1.19	.24	.11
**03. PBC: Physical Activity**	4.29 (1.59)	4.63 (1.54)	-.33	-.62	-.05	-2.29	.02	**-.21**	4.56 (1.61)	4.70 (1.46)	-.16	-.43	.11	-1.17	.25	-.11
**04. Confidence: Weight Loss**	4.33 (1.75)	5.15 (1.32)	-.90	-1.20	-.60	-5.97	<.01	**-.56**	5.00 (1.58)	5.51 (1.30)	-.43	-.69	-.18	-3.35	<.01	**-.31**
**05. Confidence: WLM**	4.08 (1.79)	4.16 (1.59)	-.14	-.44	.16	-.92	.36	-.09	5.20 (1.53)	4.30 (1.51)	.87	.56	1.18	5.52	<.01	**.51**
**06. SE: Emotional Eating**	2.56 (1.01)	2.44 (.85)	.08	-.10	.26	.87	.38	.08	2.61 (.84)	2.76 (.95)	-.12	-.29	.04	-1.45	.15	-.14
**07. SE: Unhealthy Food Context**	2.75 (.75)	2.94 (.58)	-.22	-.34	-.10	-3.72	<.01	**-.35**	2.94 (.62)	2.94 (.65)	.00	-.12	.12	.01	.99	.00
**08. SE: Physical Activity Barriers**	2.70 (.78)	2.92 (.77)	-.21	-.33	-.10	-3.66	<.01	**-.34**	2.80 (.82)	2.96 (.72)	-.16	-.30	-.02	-2.28	.02	**-.21**
**09. Action Planning: Physical Activity**	2.59 (1.11)	2.91 (1.02)	-.38	-.59	-.17	-3.56	<.01	**-.33**	2.66 (1.10)	2.83 (1.05)	-.13	-.34	.08	-1.23	.22	-.11

**10. Action Planning: Healthy Eating**	2.78 (1.00)	3.15 (.85)	-.41	-.59	-.23	-4.59	<.01	**-.43**	3.20 (.79)	3.30 (.72)	-.11	-.26	.05	-1.34	.18	-.12
**11. Coping Planning: Physical Activity**	2.06 (.92)	2.17 (.94)	-.15	-.32	.02	-1.75	.08	-.16	2.28 (1.06)	2.22 (1.01)	.08	-.13	.29	.76	.45	.07
**12. Coping Planning: Healthy Eating**	2.43 (.96)	2.62 (.90)	-.24	-.42	-.05	-2.56	.01	**-.24**	2.85 (.83)	2.81 (.88)	.03	-.14	.19	.31	.76	.03
**13. Automaticity: Healthy Eating**	2.63 (.85)	2.60 (.81)	.04	-.11	.19	.55	.58	.05	2.94 (.76)	2.74 (.81)	.18	.03	.32	2.42	.02	**.23**
**14. Automaticity: Physical Activity**	2.36 (.94)	2.36 (.88)	.02	-.11	.14	.25	.80	.02	2.49 (1.01)	2.42 (.91)	.09	-.07	.25	1.11	.27	.10
**15. Automaticity: Self-Weighing**	2.67 (1.05)	2.68 (1.02)	.00	-.21	.21	-.02	.99	.00	3.19 (.84)	2.72 (1.05)	.45	.25	.65	4.39	<.01	**.41**
**16. Energy and Drive**	3.15 (.79)	3.39 (.75)	-.24	-.35	-.14	-4.56	<.01	**-.42**	3.24 (.76)	3.51 (.70)	-.25	-.39	-.11	-3.59	<.01	**-.34**

Notes.T2 = Time 2; T1 = Time 1; SD = standard deviation; Est. = estimated difference across time points; *CI* = confidence interval; *t* = t-test statistic; *p* = p-value; *d* = Cohen's *d*; PBC = Perceived behavioral control; WLM = weight loss maintenance; SE = self-efficacy.

6. References

1. Finch EA, Linde JA, Jeffery RW, Rothman AJ, King CM, Levy RL. The effects of outcome expectations and satisfaction on weight loss and maintenance: Correlational and experimental analyses-a randomized trial. *Health Psychology*. 2005;24(6):608.
2. Schwarzer R, Renner B. *Health-specific self-efficacy scales*. 2009. Available at: http://userpage.fu-berlin.de/~health/healself.pdf
3. Clark MM, Abrams DB, Niaura RS, Eaton CA, Rossi JS. Self-efficacy in weight management. *Journal of Consulting Clinical Psychology*. 1991;59(5):739.
4. Sniehotta FF, Schwarzer R, Scholz U, Schüz B. Action planning and coping planning for long- term lifestyle change: theory and assessment. *European Journal of Social Psychology*. 2005;35(4):565-576.
5. Verplanken B, Orbell S. Reflections on past behavior: a self- report index of habit strength 1. *Journal of Applied Social Psychology*. 2003;33(6):1313-1330.
6. Gardner B, Abraham C, Lally P, de Bruijn G-J. Towards parsimony in habit measurement: Testing the convergent and predictive validity of an automaticity subscale of the Self-Report Habit Index. *International Journal of Behavioral Nutrition Physical Activity*. 2012;9(1):1-12.
